# Supplementary material for: Chemoselective single-site Earth-abundant metal catalysts at metal–organic framework nodes
Source: Nat Commun. 2016 Aug 30;7:12610. doi: 10.1038/ncomms12610 (PMC5013626; doi:10.1038/ncomms12610)
Supplement: Supplementary Information — Supplementary Figures 1-44, Supplementary Tables 1-12, Supplementary Discussion, Supplementary Methods and Supplementary References [file ncomms12610-s1.pdf]

Supplementary Figures

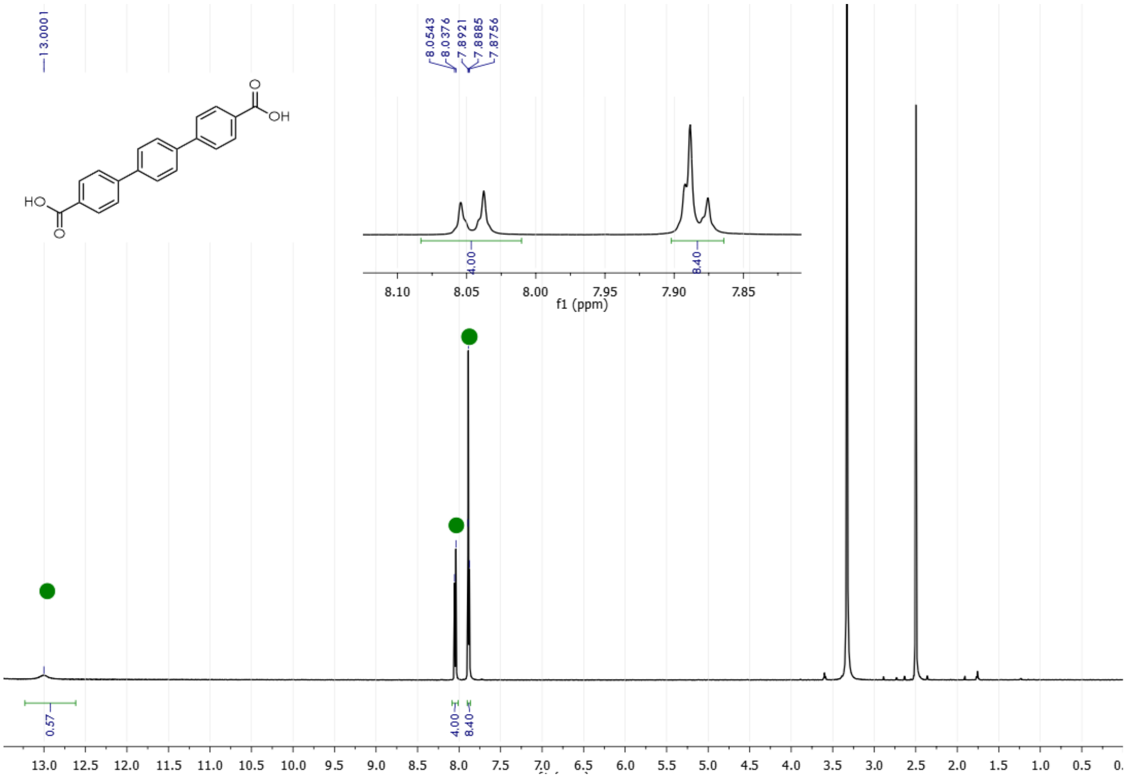

Supplementary Figure 1 / ^{1}H-NMR spectrum (500 MHz in $CDCl_3$) of 1,4-bis(4-carboxyphenyl)benzene.

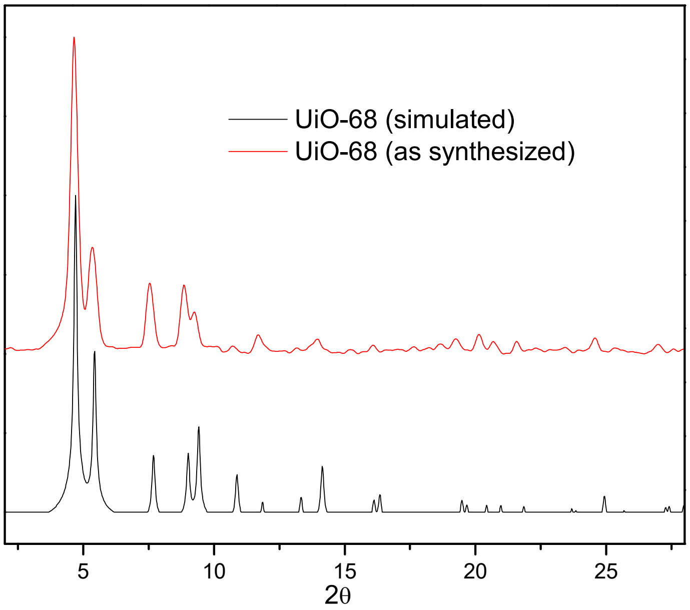

Supplementary Figure 2 / PXRD patterns of synthesized UiO-68 (red) comparing with simulated pattern from single crystal structure.

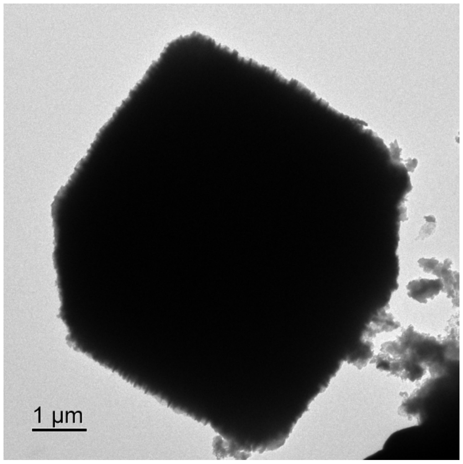

Supplementary Figure 3 / TEM image of as-synthesized UiO-68.

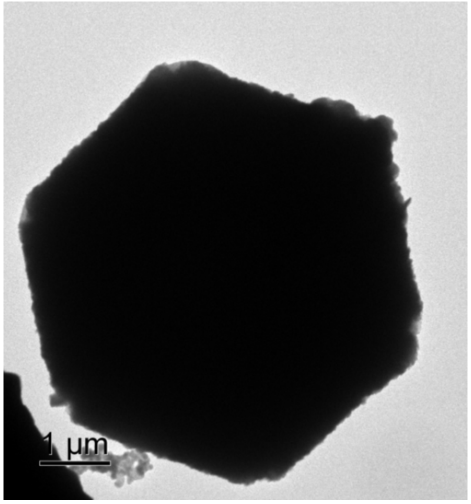

Supplementary Figure 4 / TEM image of UiO-CoCl.

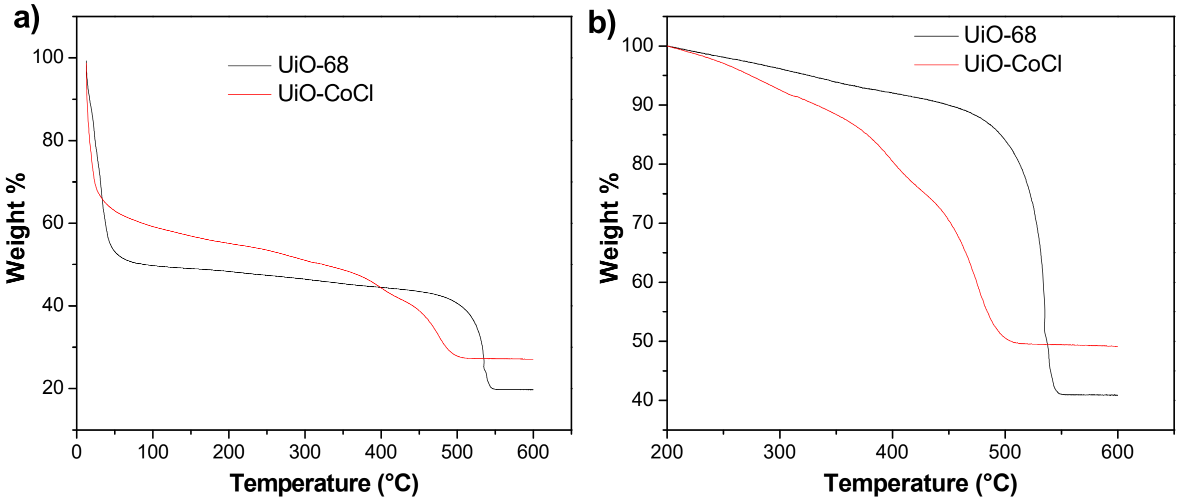

Supplementary Figure 5 / TGA curves of freshly prepared UiO-68 (black) and UiO-CoCl (red) in the 25 - 600 °C range (**a**) and 200 - 600 °C range (**b**). The increased residual mass at 600 °C is due to the presence of Co in UiO-CoCl.

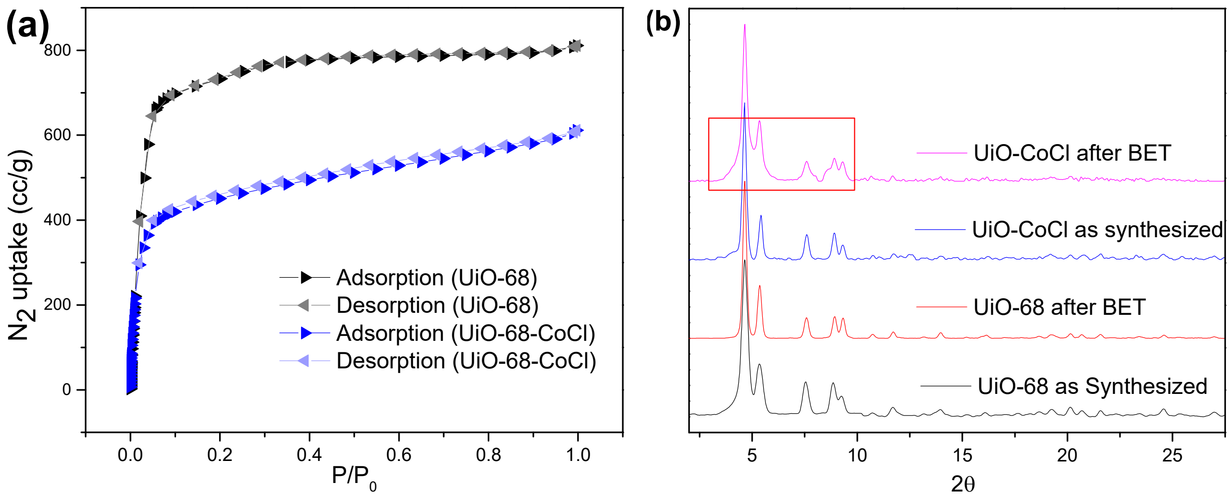

Supplementary Figure 6 / **(a)** Nitrogen sorption isotherms of UiO-68 (77 K) and UiO-CoCl (77 K). UiO-68 and UiO-CoCl have BET surface areas of 2815 m^2/g and 1815 m^2/g, respectively. **(b)** The slightly lower surface area of UiO-CoCl is due to the increase of molecular weight after metalation (15%) and reduced pore sizes due to the presence of CoCl species (by ~15%). The expected surface area for UiO-CoCl would be ~2815/(1.15×1.15)=2129 m^2/g. The lightly smaller experimental value of 1815 m^2/g is likely due to distortion of the framework upon removal of the solvent, as suggested by the broadening of the PXRD pattern of UiO-CoCl after BET analysis.

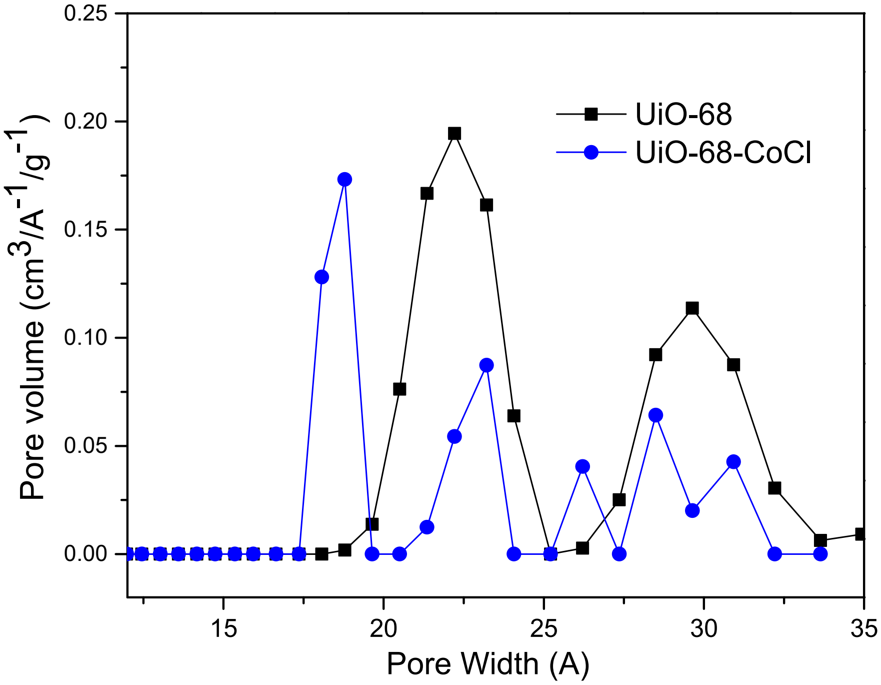

Supplementary Figure 7 / Pore size distributions of UiO-68 (black) and UiO-CoCl (red) with a non-local density functional theory (NLDFT).

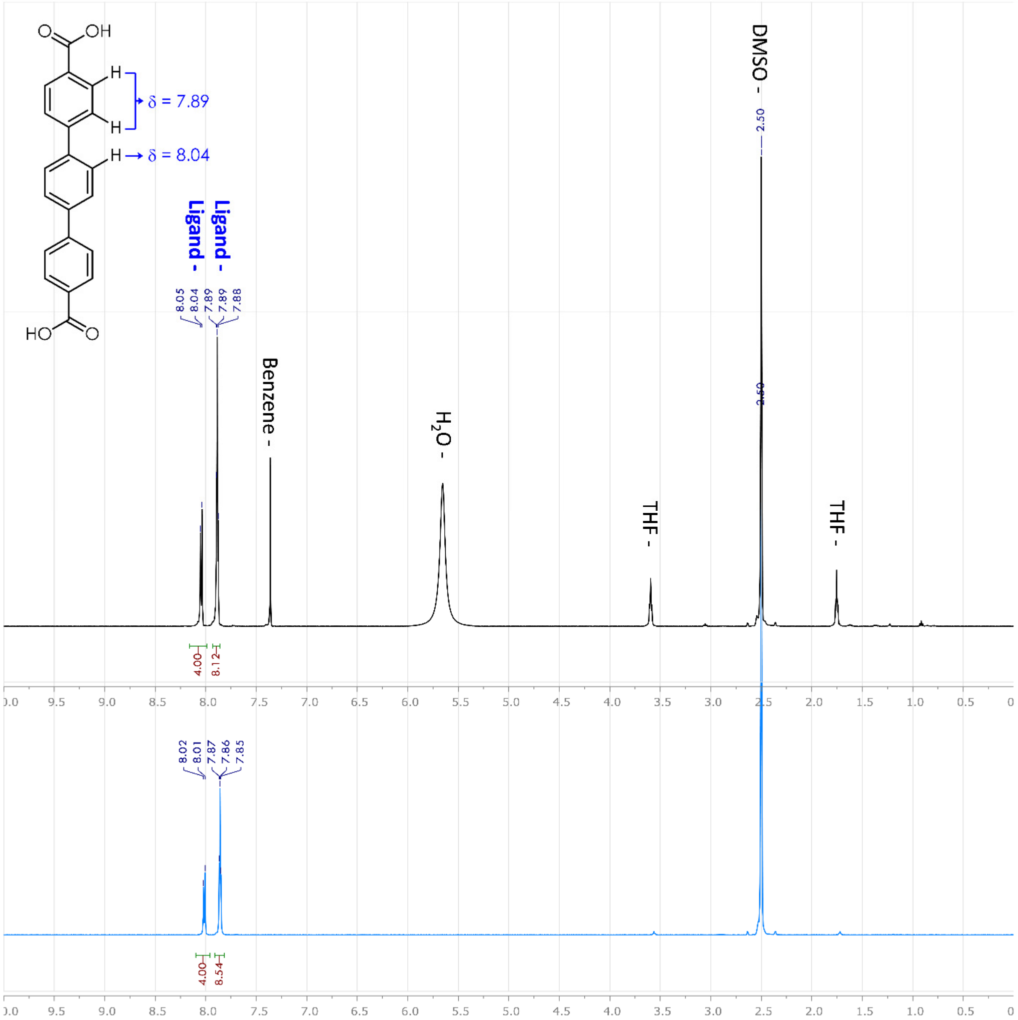

Supplementary Figure 8 / ^{1}H NMR (500 MHz) spectra of digested lithiated UiO-68 (black) compared with spectra of pure ligand (blue). 5% D_2SO_4 in DMSO-d_6 was used as ^{1}H NMR solvent. ^{1}H NMR spectrum of the digested lithiated UiO-68-MOF showed that the carboxylates of UiO-68-MOF were remained intact after treatment of nBuLi for metalation.

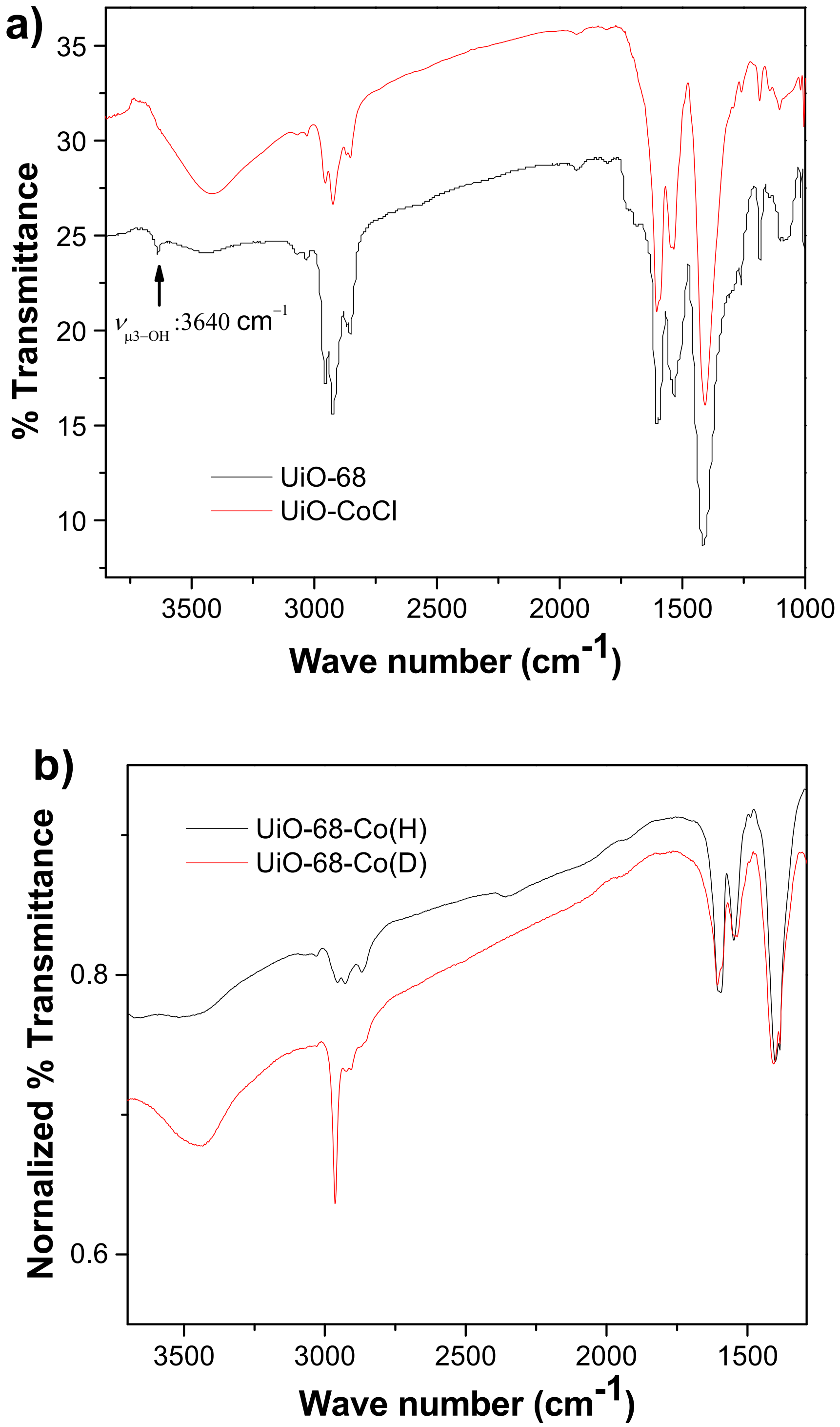

Supplementary Figure 9 / (a) IR spectra of UiO-68 (black) and UiO-CoCl (red). The disappearance of $\nu_{\mu3O\text{-}H}$ band (~3640 cm^{-1}, KBr) suggests post-synthetic metalation at Zr_3-μ_3-OH sites. Each sample was dried under vacuum at 100 °C overnight before sample preparation. **(b)** IR spectra of UiO-CoH (black) and UiO-CoD (red). We assigned the weak peak at ~2350 cm^{-1} to the Co-H stretching, which disappeared in the UiO-CoD analog. The even weaker Co-D stretching peak is expected to be buried under the strong carboxylate stretching at ~1662 cm^{-1}.

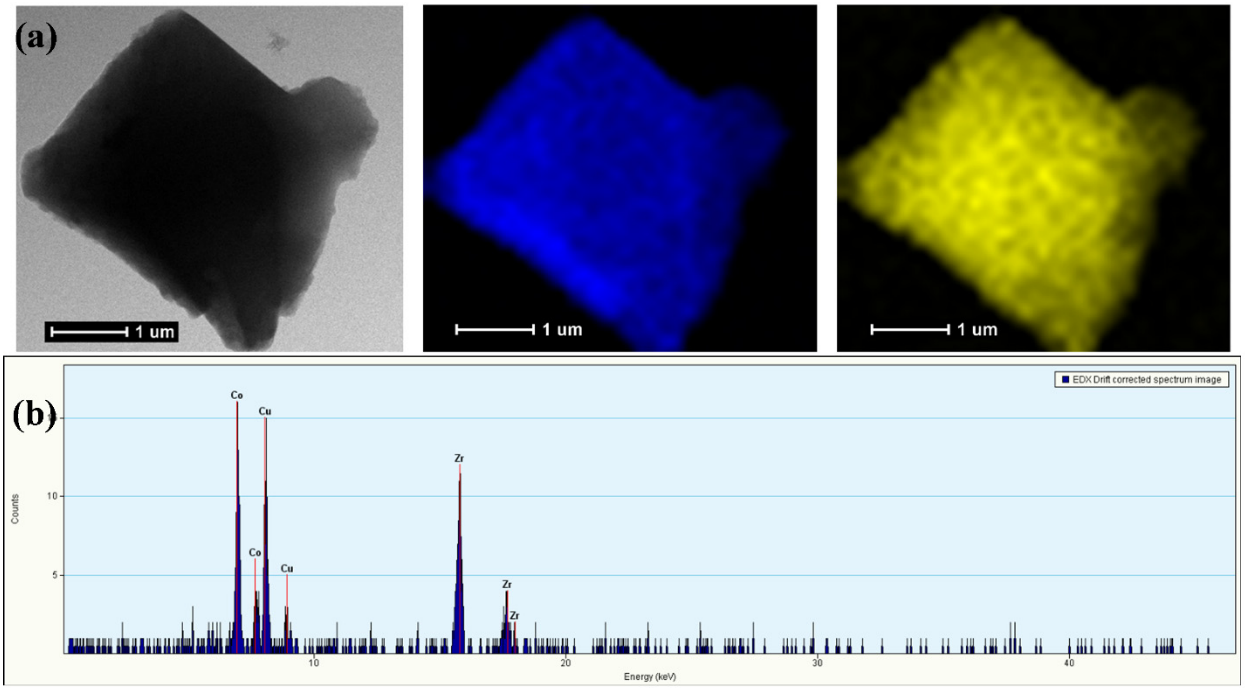

Supplementary Figure 10 / (a) TEM-EDX Mapping of UiO-Co: TEM Image (left), Co distribution is shown in blue (middle), and Zr distribution is shown in yellow (right). TEM-EDX mapping of UiO-Co indicates that Co and Zr are well-dispersed throughout the MOF-particle. **(b)** EDX analysis of the whole particle.

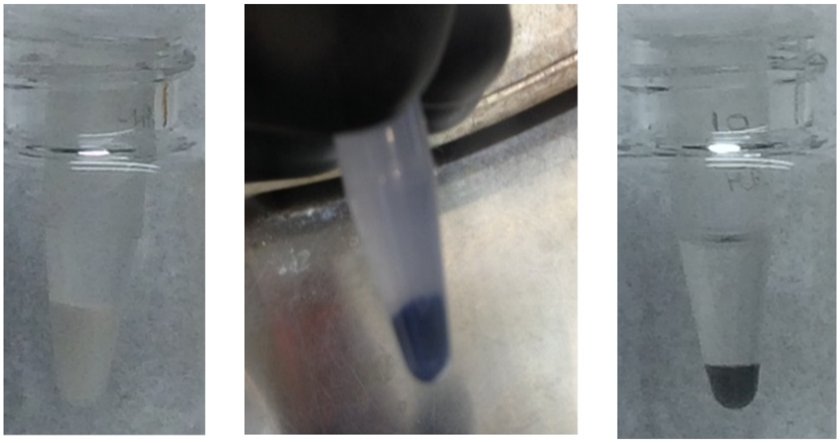

Supplementary Figure 11 / Photos showing the UiO-68 as white solid (left), UiO-CoCl as deep blue solid (middle), and UiO-FeBr as deep brown solid (right).

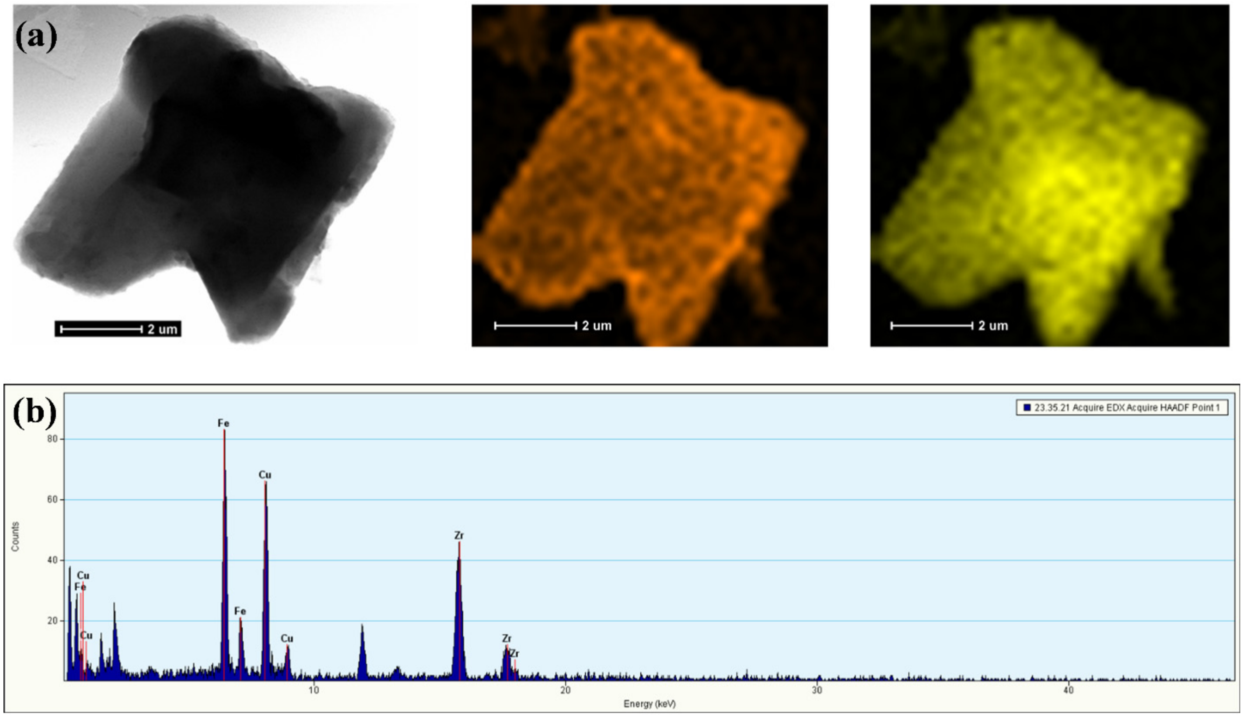

Supplementary Figure 12 / (a) TEM-EDX Mapping of UiO-Fe: TEM Image (left), Fe distribution is shown in orange (middle), and Zr distribution is shown in yellow (right). TEM-EDX mapping of UiO-Fe indicates that Fe and Zr are well-dispersed throughout the MOF-particle. **(b)** EDX analysis of the whole particle.

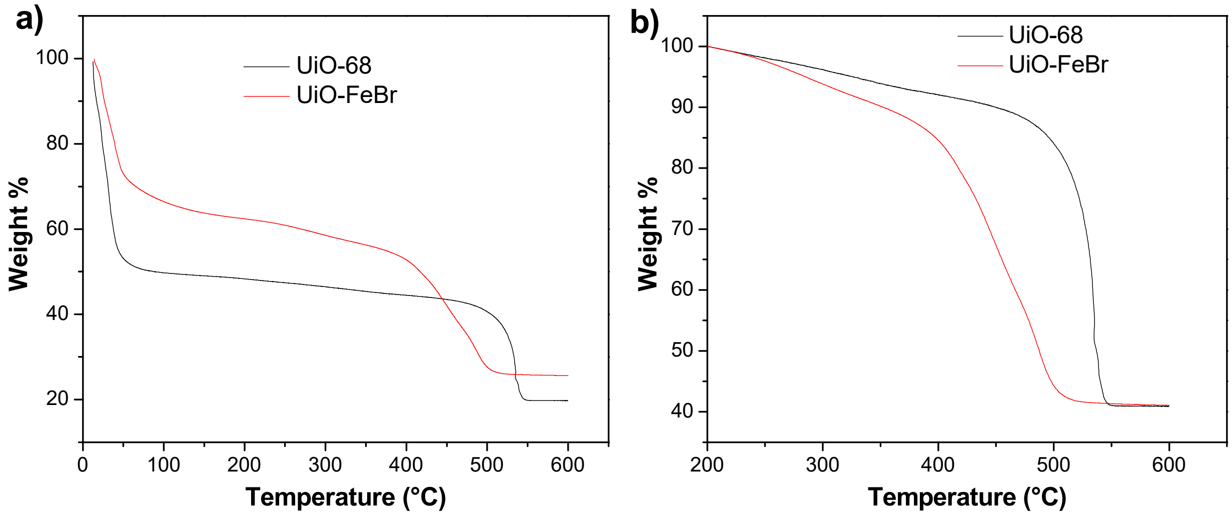

Supplementary Figure 13 / TGA curves of freshly prepared UiO-68 (black) and UiO-FeBr (red) in the 25 - 600 °C range (**a**) and 200 - 600 °C range (**b**). The increased residual mass at 600 °C is due to the presence of Fe in UiO-FeBr.

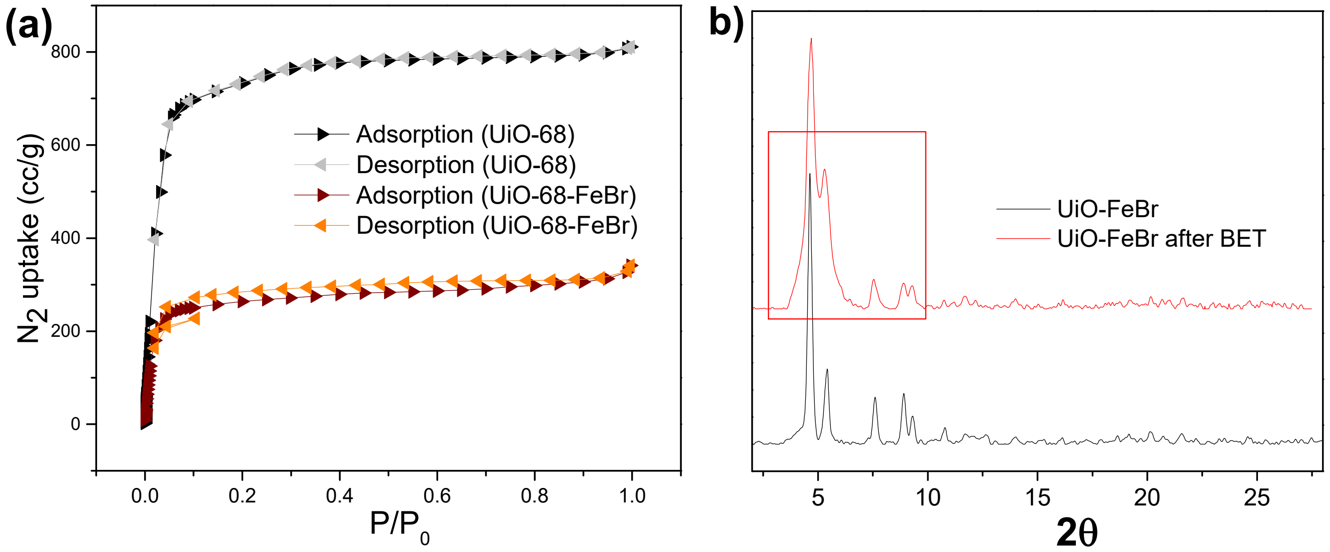

Supplementary Figure 14 / Nitrogen sorption isotherms of UiO-68 (77 K) and UiO-FeBr (77 K). UiO-68 and UiO-FeBr have BET surface areas of 2815 m^2/g and 1160 m^2/g, respectively. **(b)** The low surface area of UiO-FeBr is due to the increase in molecular weight of MOF after metalation (by 21%) and reduced pore sizes due to the presence of FeBr species (by ~21%). The expected surface area for UiO-FeBr would be ~2815/(1.21×1.21)=1923 m^2/g. The lightly smaller experimental value of 1160 m^2/g is likely due to distortion of the framework upon removal of the solvent, as suggested by the broadening of the PXRD pattern of UiO-FeBr after BET analysis.

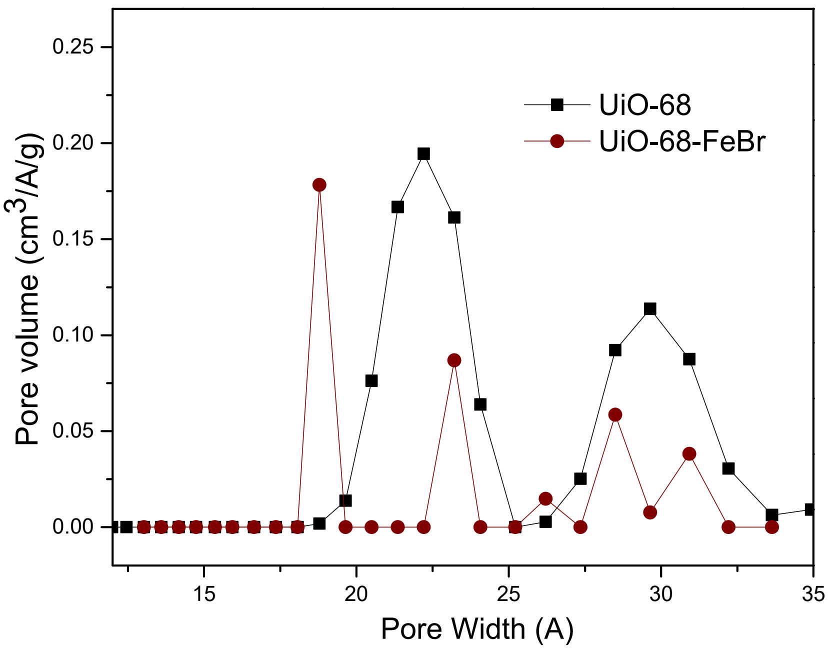

Supplementary Figure 15 / Pore size distributions of UiO-68 (black) and UiO-FeBr (red) with a non-local density functional theory (NLDFT).

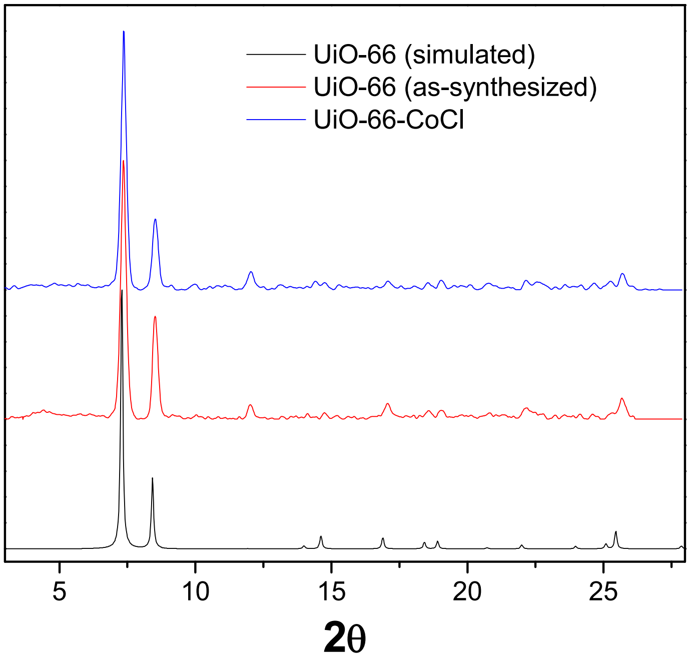

Supplementary Figure 16 / Similarities between the PXRD pattern simulated from the CIF file of UiO-66 (black) and the experimental PXRD patterns of UiO-66 (green) and UiO-66-CoCl indicate the retention of UiO-66 crystallinity after postsynthetic metalation.

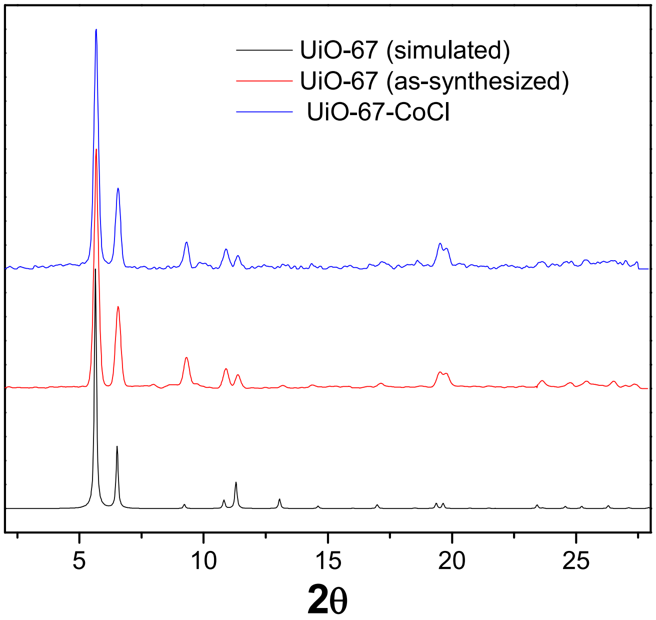

Supplementary Figure 17 / Similarities between the PXRD pattern simulated from the CIF file of UiO-67 (black) and the experimental PXRD patterns of UiO-67 (green) and UiO-67-CoCl indicate the retention of UiO-67 crystallinity after postsynthetic metalation.

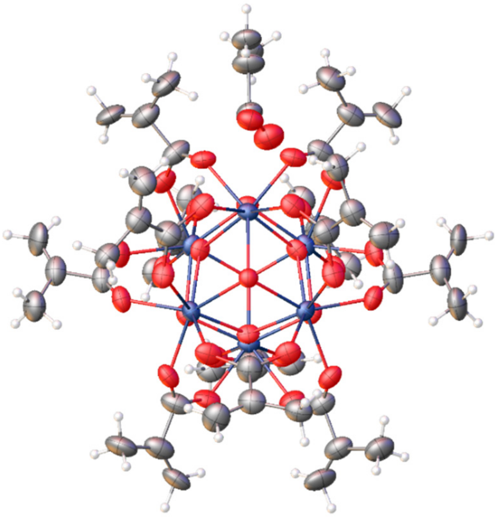

Supplementary Figure 18 / Single crystal structure of homogenous $Zr_6O_4(OH)_4(McO)_{12}$ cluster.

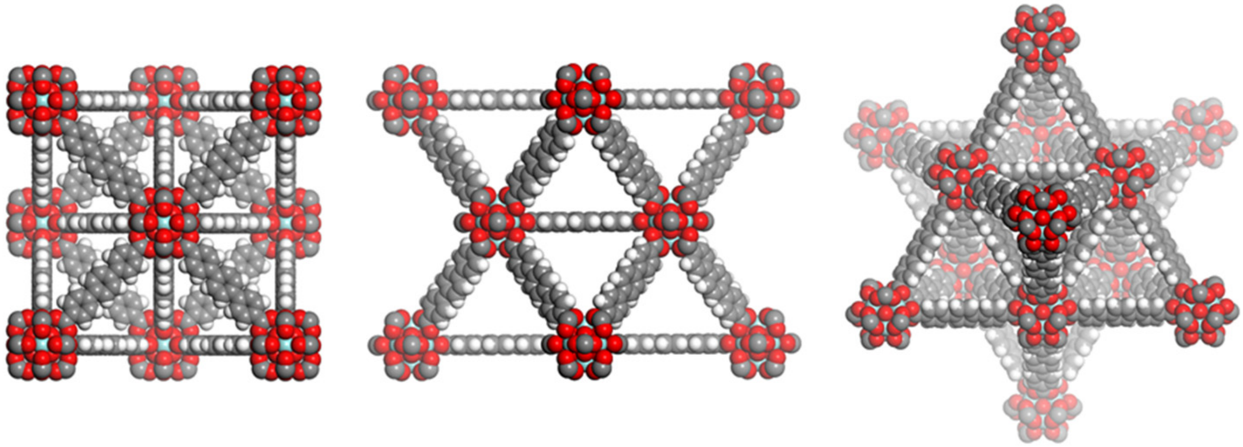

Supplementary Figure 19 / Space-filling models of UiO-CoCl, as viewed along the [100] (top left), the [110] (top right), and the [111] (bottom) directions. The positions of cobalt or chloride cannot be determined due to the intrinsic disorders as shown in Supplementary Fig 20.

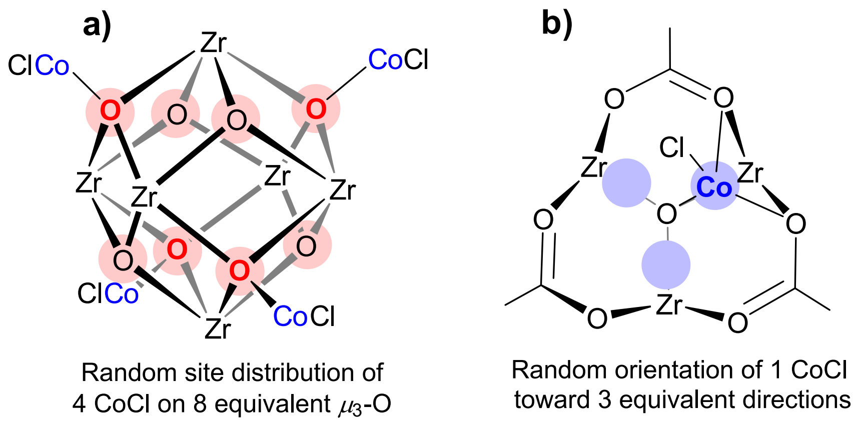

Supplementary Figure 20 / The loading of CoCl to μ_3-O exhibits site disorder because of the random distribution of μ_3-OH in the Zr_6 SBU (a) and the orientation disorder of CoCl, even when bound to the same μ_3-O (b). Both kinds of disorder are present in the homogeneous Zr_6 cluster, as well, making it impossible to determine its crystal structure.

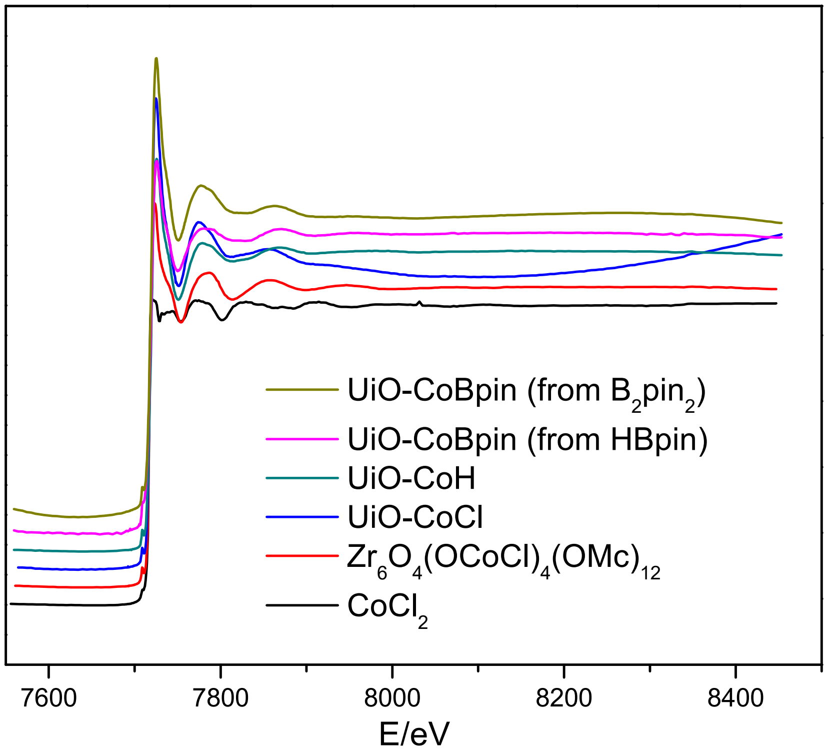

Supplementary Figure 21 / Normalized μ(E) XAS spectra for the $CoCl_2$ reference compound, $Zr_6O_4(OCoCl)_4(OMc)_{12}$, UiO-CoCl, UiO-CoH, UiO-CoBpin (prepared from HBpin), and UiO-Co-Bpin (prepared from B_2pin_2).

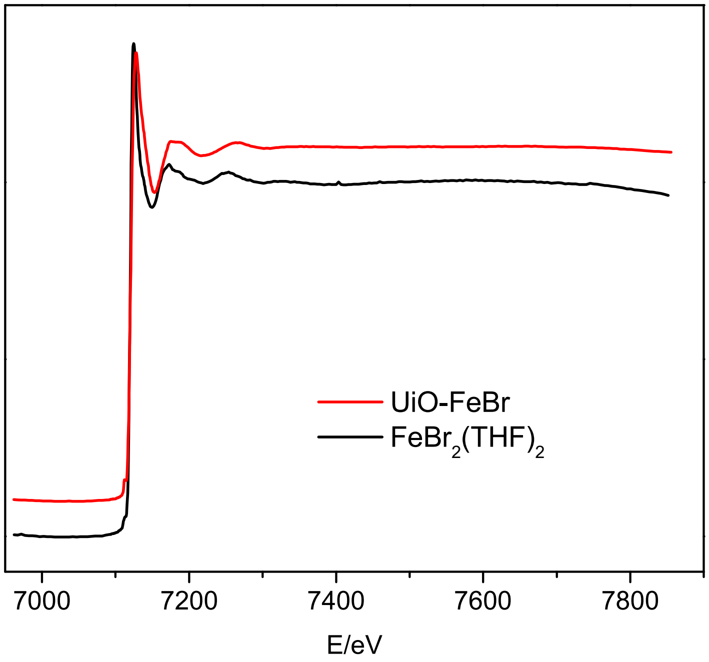

Supplementary Figure 22 / Normalized μ(E) XAS spectra for the $FeBr_2(THF)_2$ reference compound and UiO-FeBr.

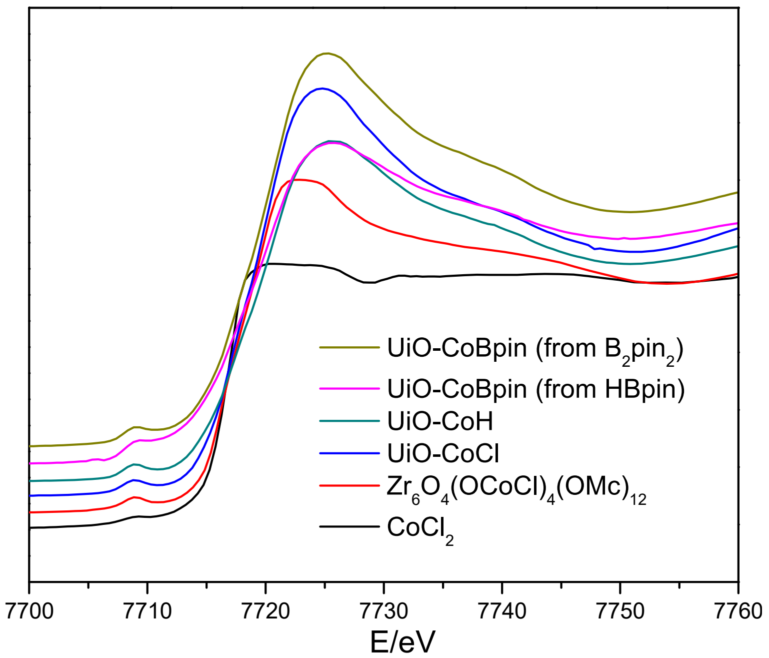

Supplementary Figure 23 / XANES spectra of the $CoCl_2$ reference compound, $Zr_6O_4(OCl)_4(OMc)_{12}$, UiO-CoCl, UiO-CoH, UiO-CoBpin (prepared from HBpin), and UiO-Co-Bpin (prepared from B_2pin_2). The oxidation states of Co species loaded into UiO-68 or $Zr_6O_4(OCl)_4(OMc)_{12}$ homogeneous clusters were determined by comparing the energies of the pre-edge peaks to the $CoCl_2$ reference compound. The positions of the pre-edge peaks align well with that of the $CoCl_2$ reference compound, which is 7708.86 eV. Therefore, we assign the oxidation state of +2 to the cobalt in all the Co-functionalized MOF materials.

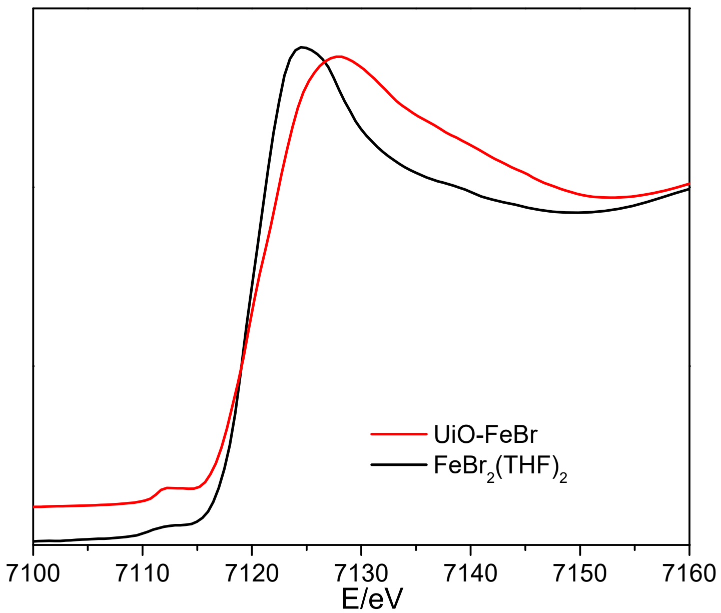

Supplementary Figure 24 / XANES spectra of $FeBr_2(THF)_2$ reference compound and UiO-FeBr. The position of the pre-edge peak of UiO-FeBr aligns well with that of $FeBr_2$ reference compound, which is 7712.13 eV. Therefore, we assign the oxidation state of +2 to the iron in UiO-FeBr.

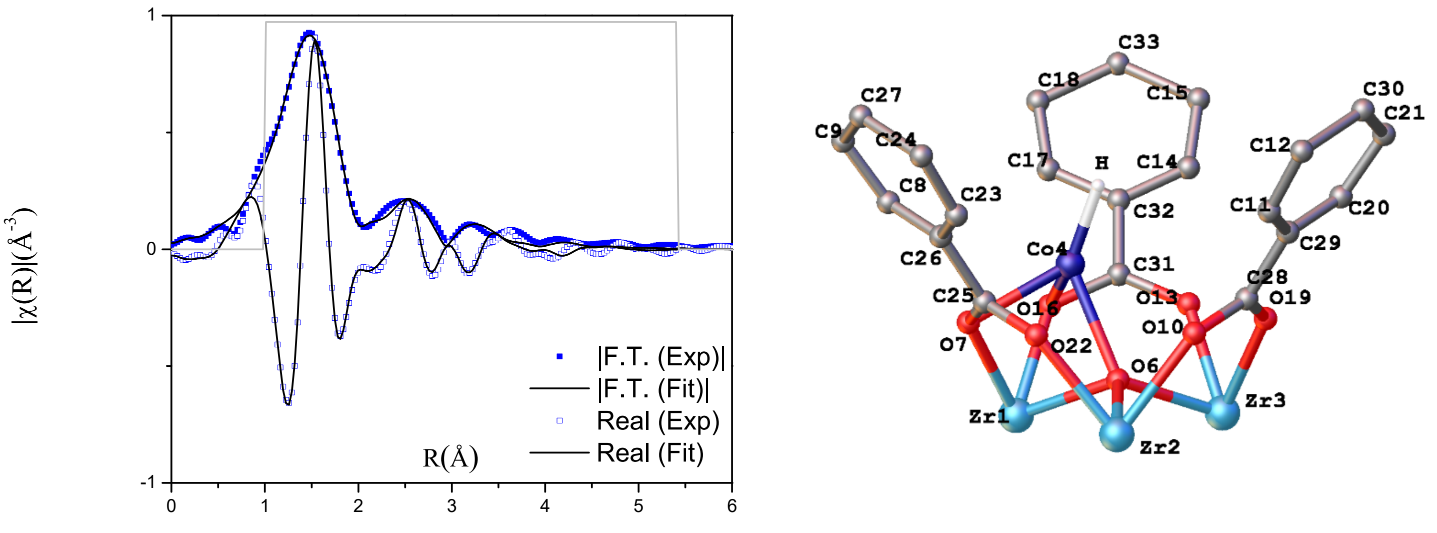

Supplementary Figure 25 / Experimental EXAFS spectra and fits of UiO-CoH in R space showing the magnitude of Fourier Transform (solid squares, solid line) and real components (hollow squares, dashed line) (left). The fitting range is 1 – 5.4 Å in R space (within the gray solid lines). The model used for EXAFS fitting, which is generated from Material Studio 7.0, is based on the crystal structure of UiO-68 (right).

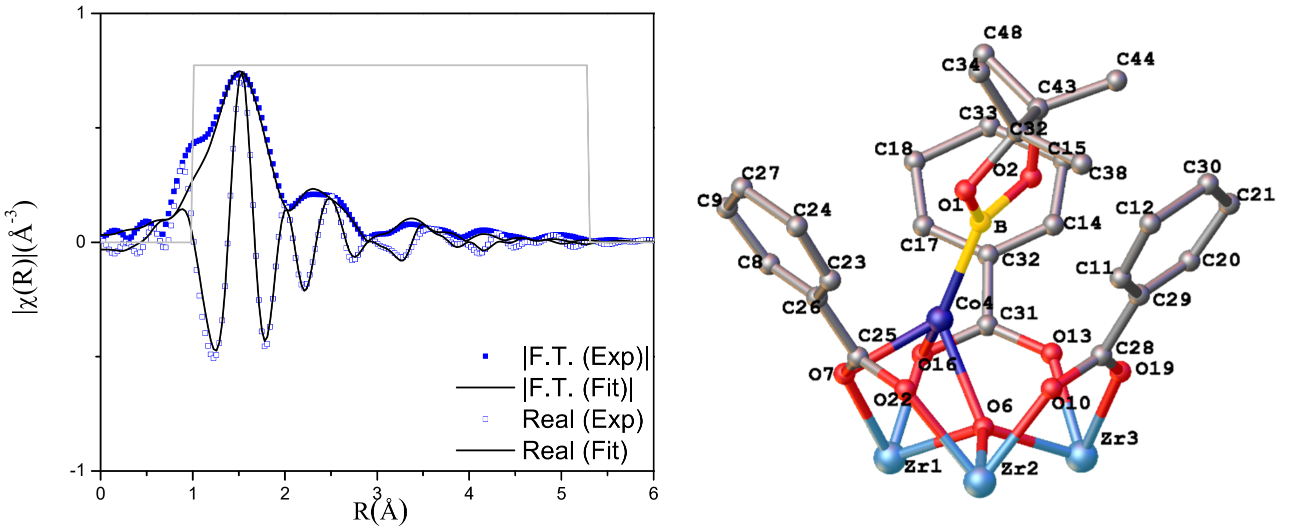

Supplementary Figure 26 / Experimental EXAFS spectra and fits of UiO-Co-Bpin (treated with HBpin) in R space showing the magnitude of Fourier Transform (solid squares, solid line) and real components (hollow squares, dashed line). The fitting range is 1 – 5.3 Å in R space). The fitting range is 1 – 5.3 Å in R space (within the gray solid lines). The model used for EXAFS fitting, which is generated from Material Studio 7.0, is based on the crystal structure of UiO-68 (right).

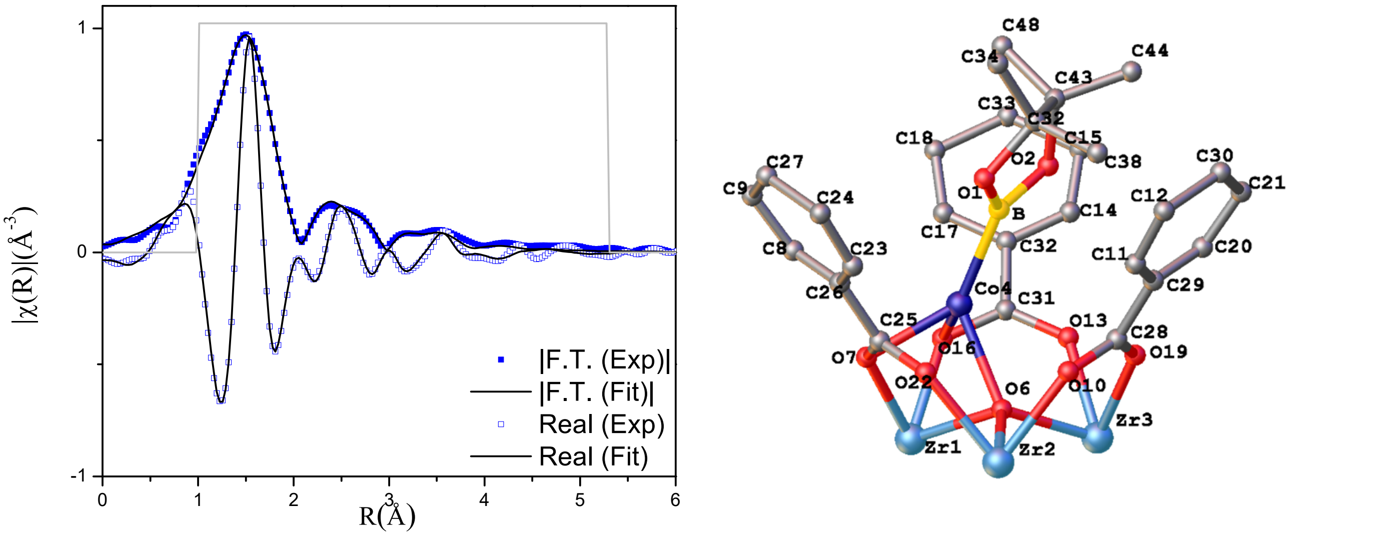

Supplementary Figure 27 / Experimental EXAFS spectra and fits of UiO-Co-Bpin (treated with B_2Pin_2) in R space showing the magnitude of Fourier Transform (solid squares, solid line) and real components (hollow squares, dashed line). The fitting range is 1 – 5.3 Å in R space (within the gray solid lines) (left). The model used for EXAFS fitting, which is generated from Material Studio 7.0, is based on the crystal structure of UiO-68 (right).

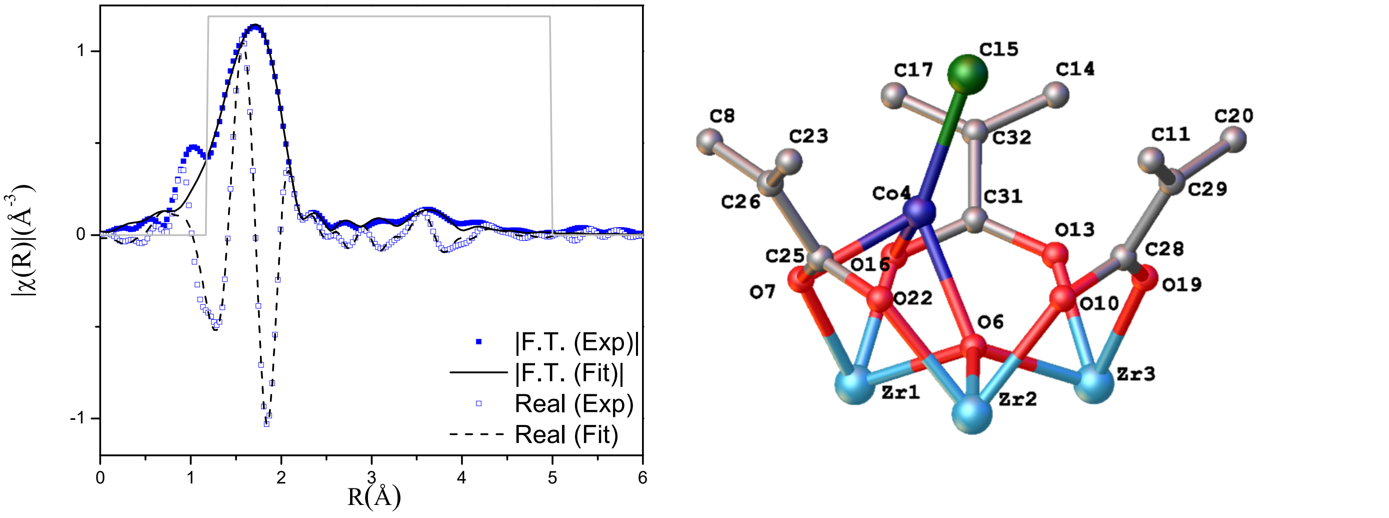

Supplementary Figure 28 / Experimental EXAFS spectra and fits of $Zr_6(OCoCl)_4O_4(OMc)_{12}$ in R space showing the magnitude of Fourier Transform (solid squares, solid line) and real components (hollow squares, dashed line). The fitting range is 1.18 – 4.98 Å in R space (within the gray solid lines) (left). The model used for EXAFS fitting, which is generated from Material Studio 7.0, is based on the crystal structure of UiO-68 (right).

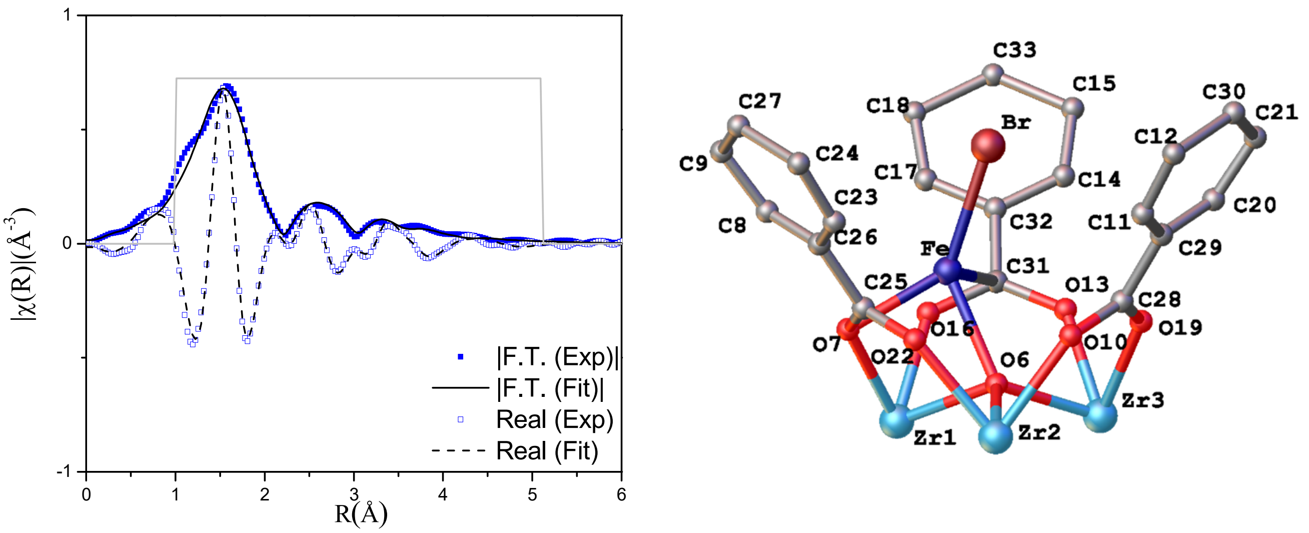

Supplementary Figure 29 / Experimental EXAFS spectra and fits of UiO-FeBr in R space showing the magnitude of Fourier Transform (solid squares, solid line) and real components (hollow squares, dashed line). The fitting range is 1 – 5.1 Å in R space (within the gray solid lines) (left). The model used for EXAFS fitting, which is generated from Material Studio 7.0, is based on the crystal structure of UiO-68 (right).

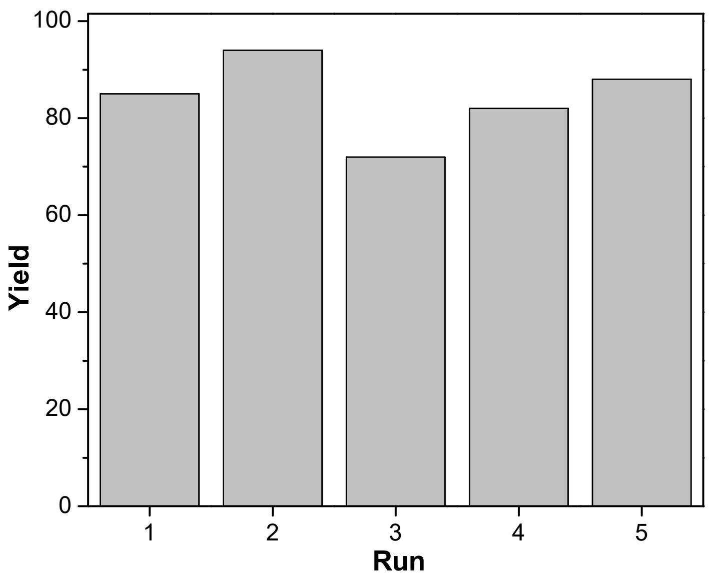

Supplementary Figure 30 / Plots of yields (%) of boronate ester at different runs in the reuse experiments of UiO-Co for benzylic C–H borylation of *p*-xylene at 98 °C. The Co loadings were 1.0 mol%.

Model for explaining selective borylation of benzylic C-H over aryl C-H groups

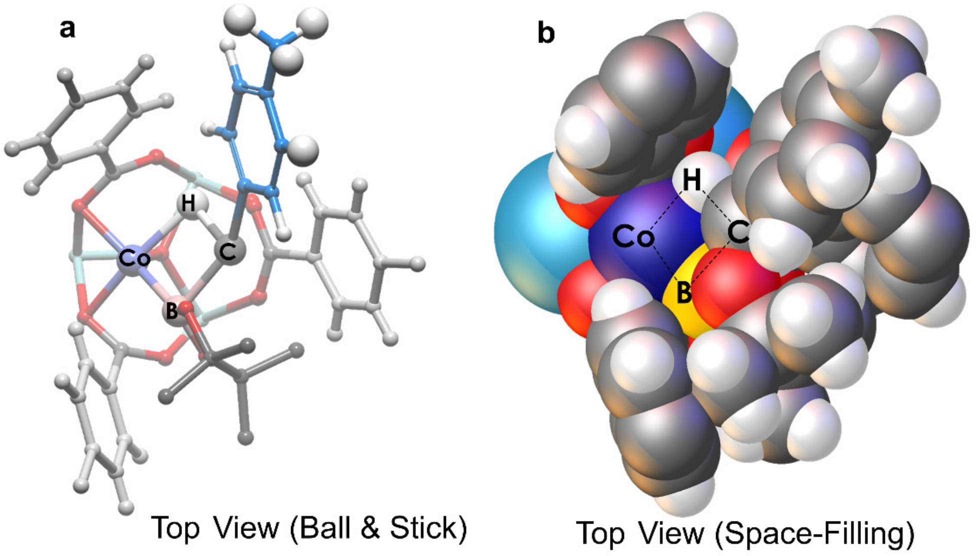

Supplementary Figure 31 / Top view of σ-bond metathesis intermediate shown in ball and stick model (a) and space filling model (b). The steric hindrance of phenyl group disfavors the approach of the aryl group to the Co site. The TPDC ligands can rotate to allow the benzylic C-H bond to approach the Co center.

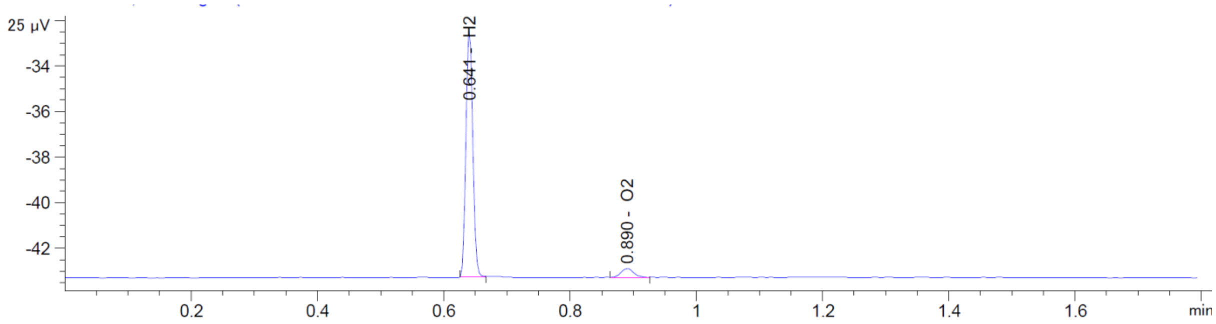

Supplementary Figure 32 / GC trace of the headspace gas. (O_2 contaminant comes from trapped air between J. Young Tube cap and rubber stopper).

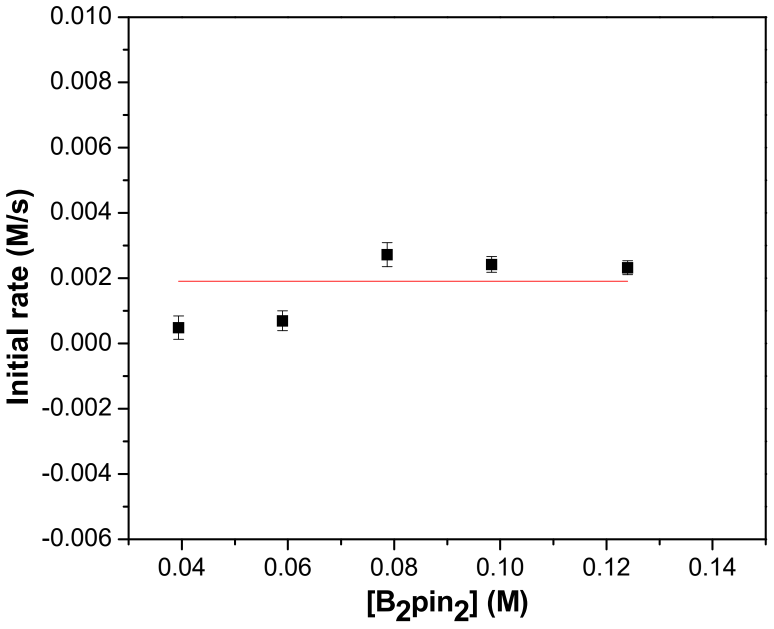

Supplementary Figure 33 / Plot of initial rate (d[*p*-xylene]/d*t*) versus $[B_2pin_2]_{initial}$ for first 12 h (<10% conversion) showing the independence of initial rates on the B_2pin_2 concentrations. [Co] = 2.0×10^{-4} M; [*p*-xylene] = 3.41×10^{-1} M.

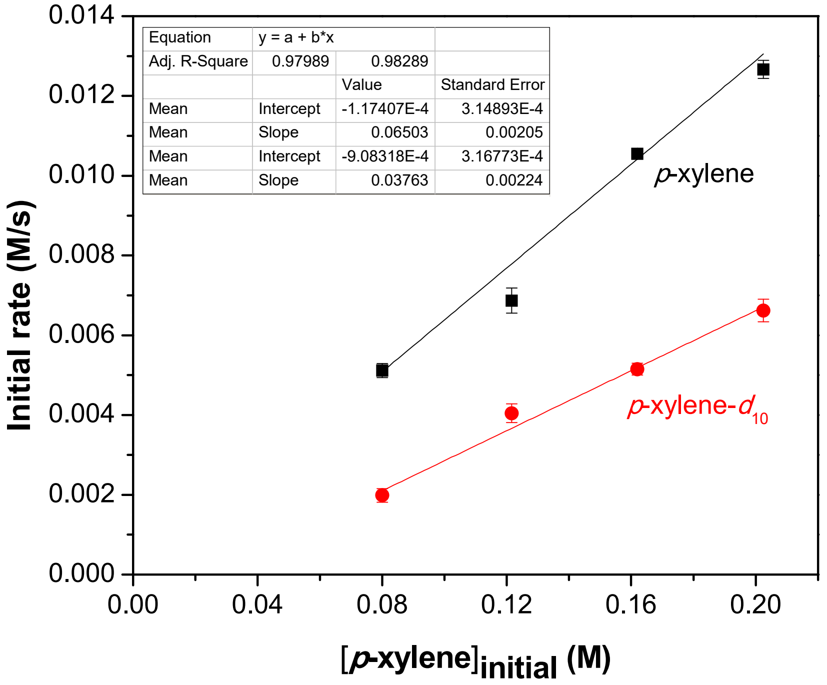

Supplementary Figure 34 / Plot of initial rates (d[*p*-xylene]/d*t*) versus $[p\text{-xylene}]_{initial}$ (<10% conversion) for benzylic C–H borylation of *p*-xylene and *p*-xylene-d_{10} at 103 °C catalyzed by UiO-Co. From the slopes of the two curves, k_H/k_D = 1.73(9).

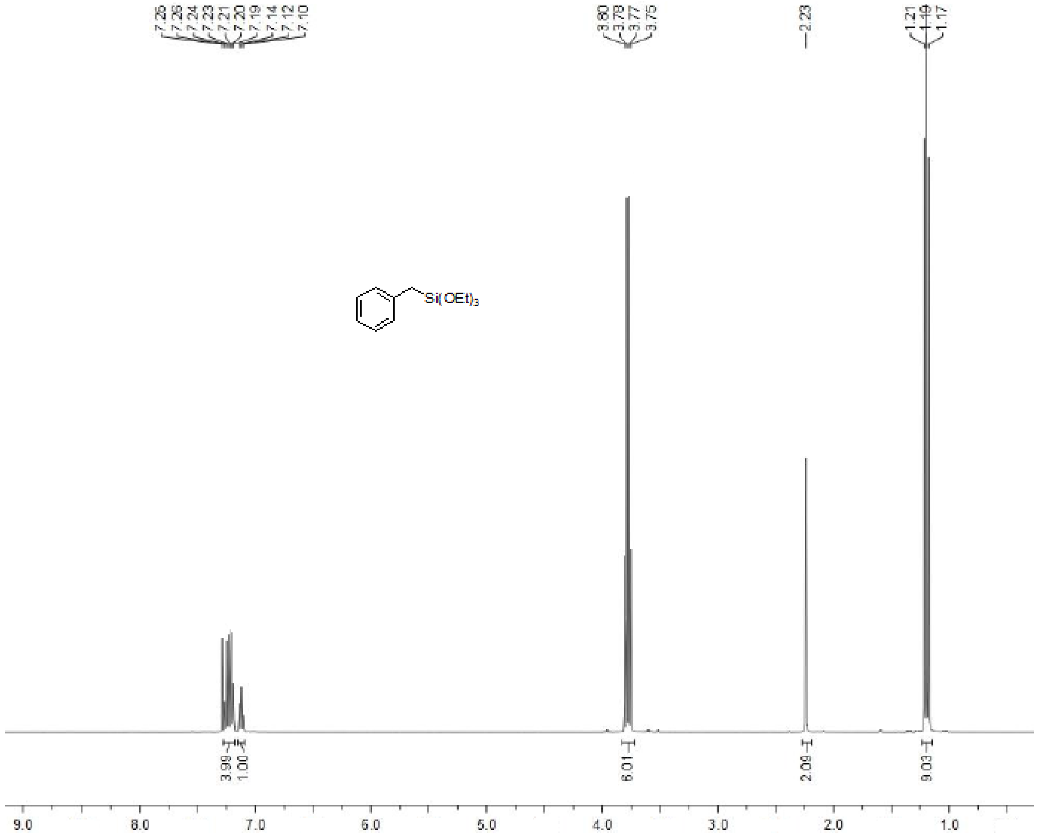

Supplementary Figure 35 / ^{1}H NMR spectrum (400 MHz, $CDCl_3$) of benzyltriethoxysilane.

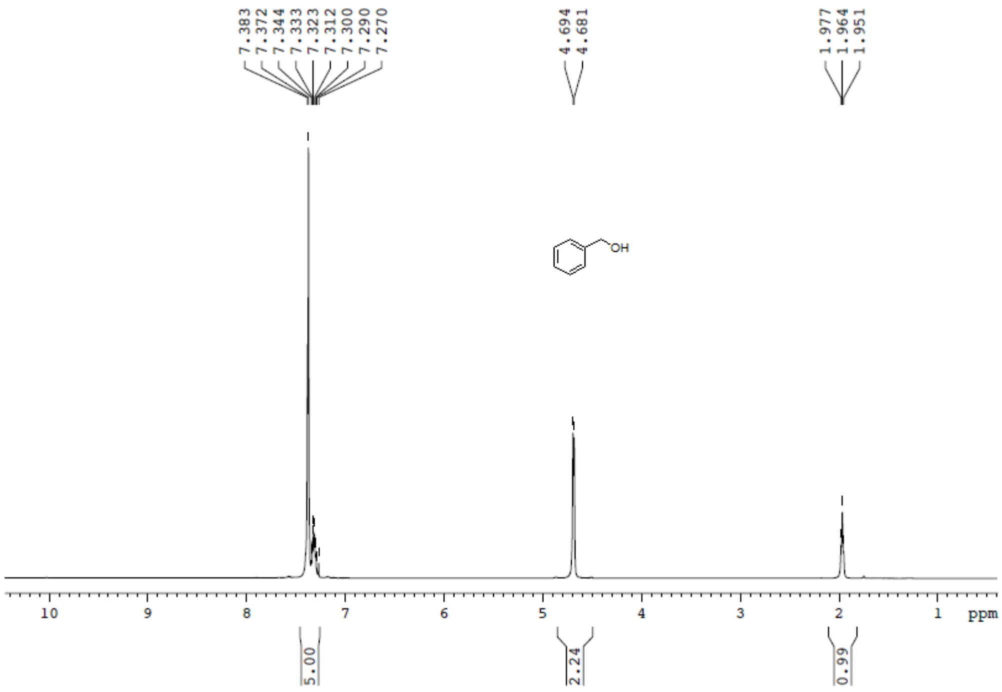

Supplementary Figure 36 / ^{1}H NMR spectrum (400 MHz, $CDCl_3$) of benzyl alcohol.

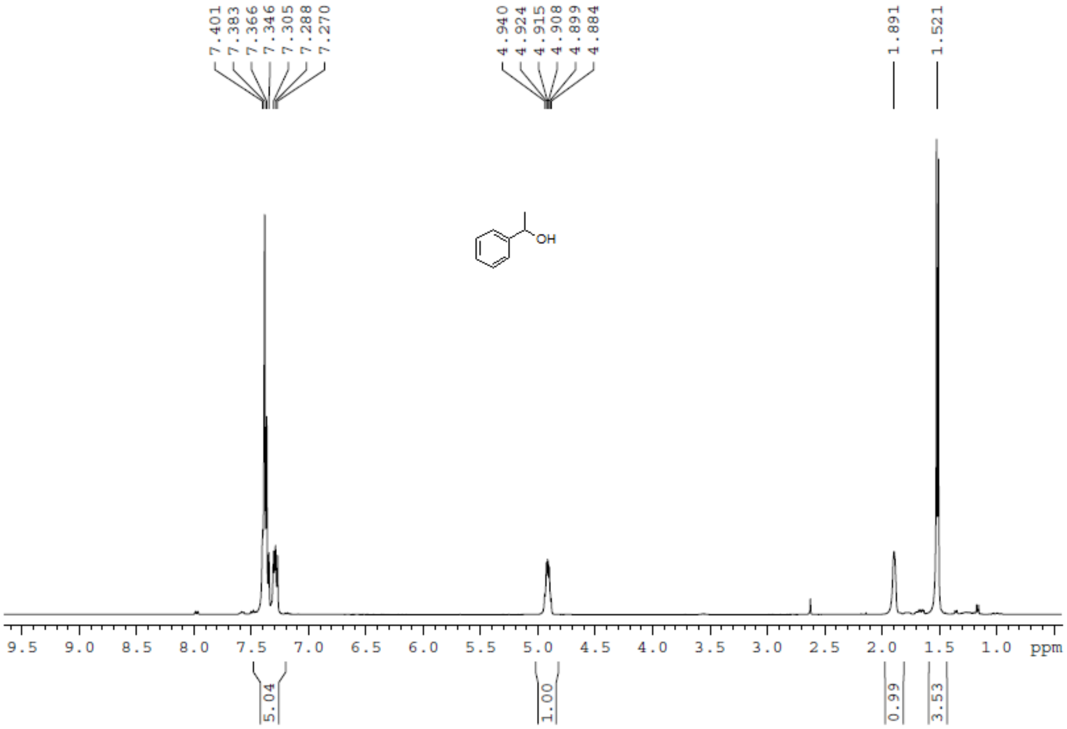

Supplementary Figure 37 / ^{1}H NMR spectrum (400 MHz, $CDCl_3$) of 1-phenylethanol.

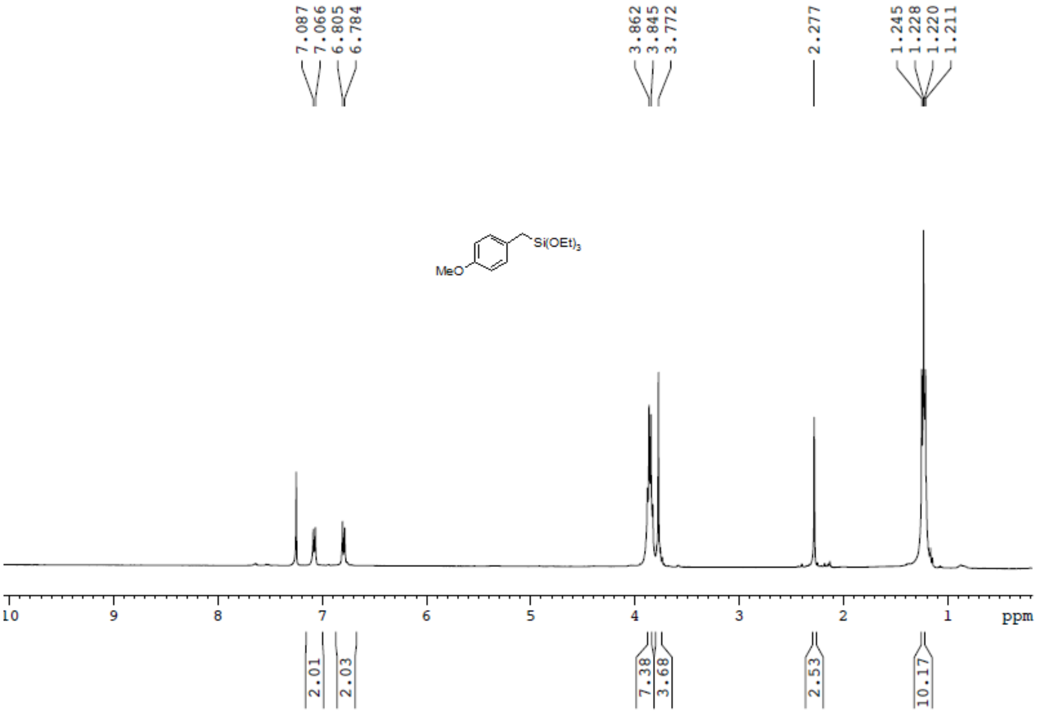

Supplementary Figure 38 / ^{1}H NMR spectrum (400 MHz, $CDCl_3$) of 4-methoxybenzyltriethoxysilane.

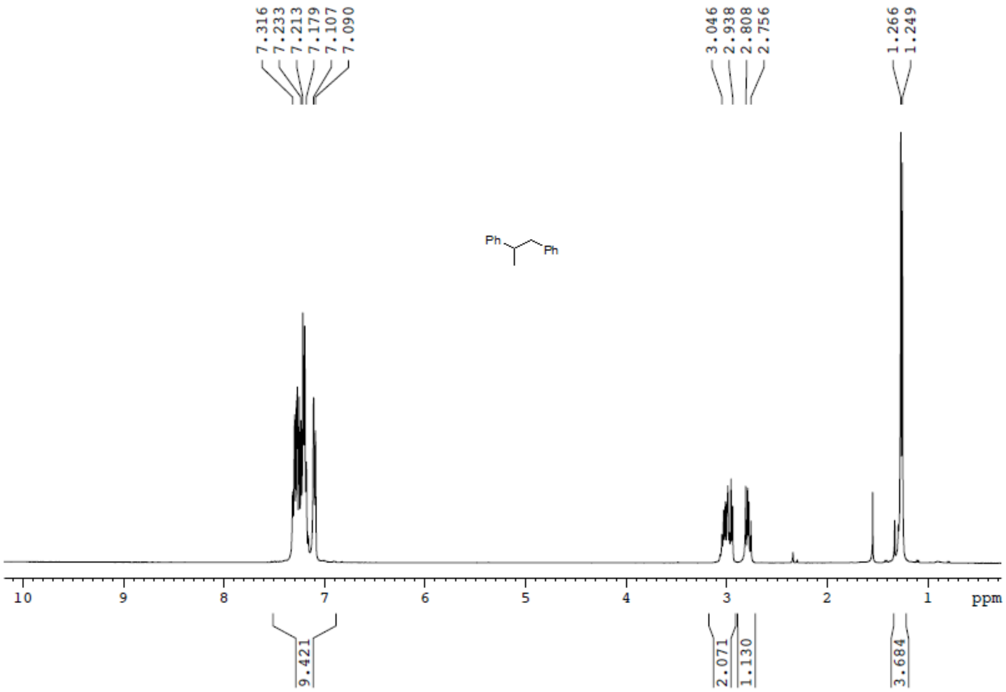

Supplementary Figure 39 / ^{1}H NMR spectrum (400 MHz, $CDCl_3$) of crude 1,2-diphenylpropane.

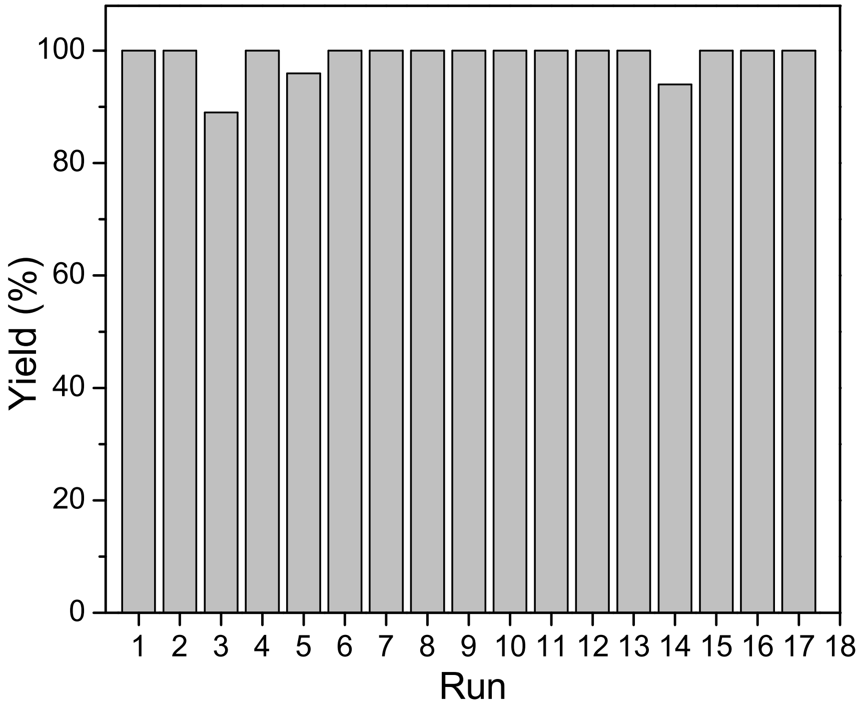

Supplementary Figure 40 / Plots of yields (%) of *n*-octane at different runs in the reuse experiments of UiO-Co for hydrogenation of 1-octene. The Co loadings were 0.01 mol%.

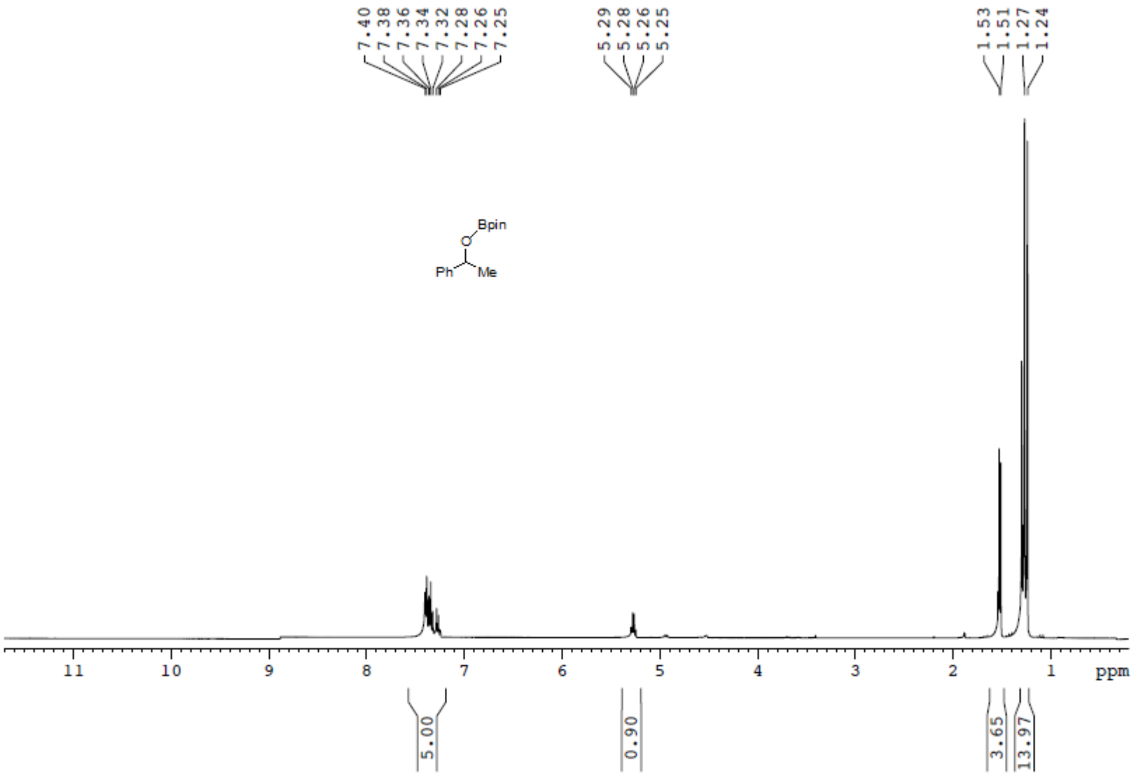

Supplementary Figure 41 / ^{1}H NMR spectrum (400 MHz, $CDCl_3$) of crude borate ester product obtained from hydroboration of acetophenone.

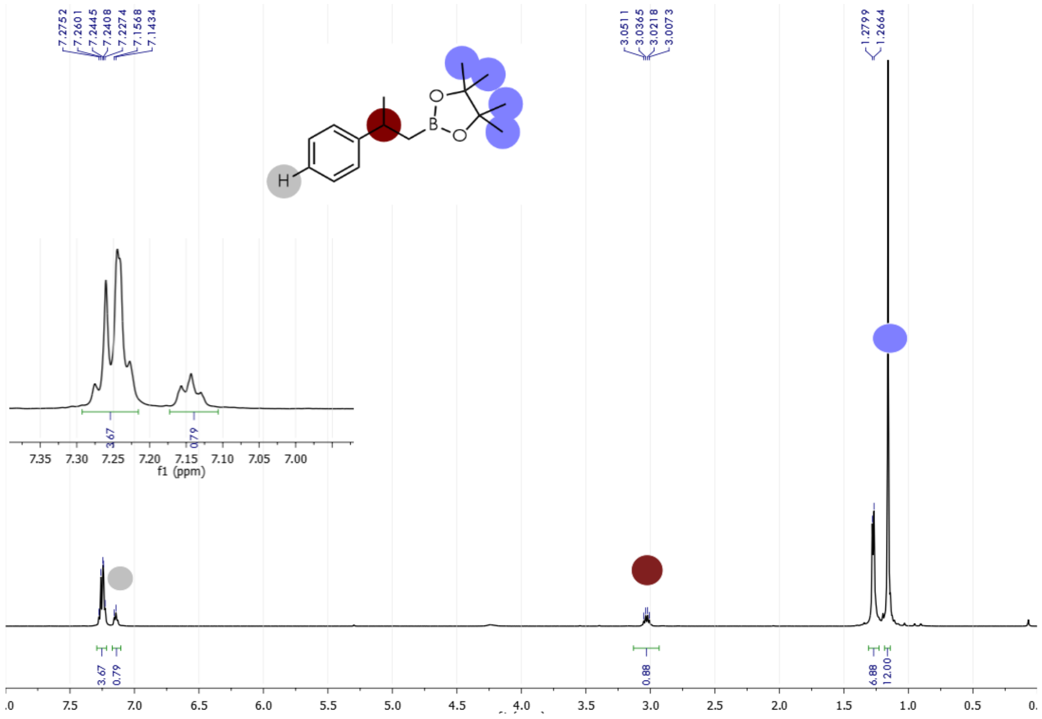

Supplementary Figure 42 / ^{1}H NMR spectrum (400 MHz, $CDCl_3$) of crude alkyl borate ester product obtained from hydroboration of α-methylstyrene.

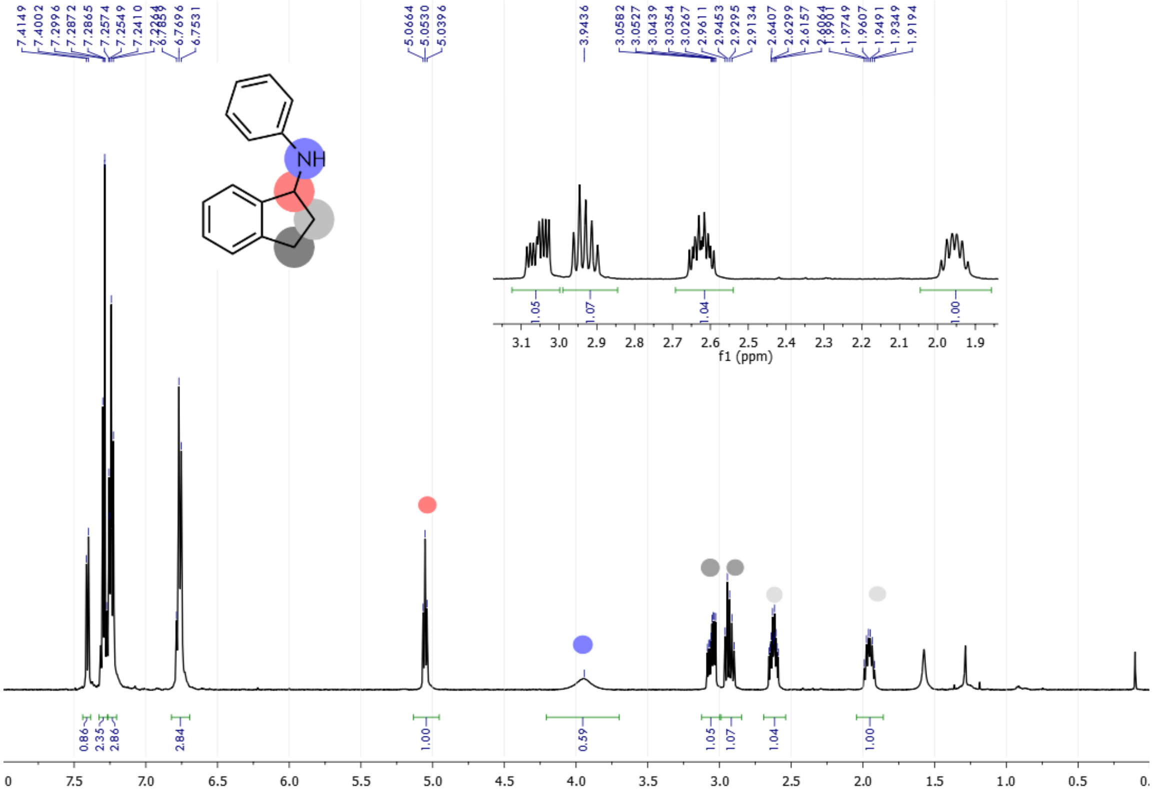

Supplementary Figure 43 / ^{1}H NMR spectrum (500 MHz, $CDCl_3$) of isolated amination product obtained from C-H amination of indane with aniline.

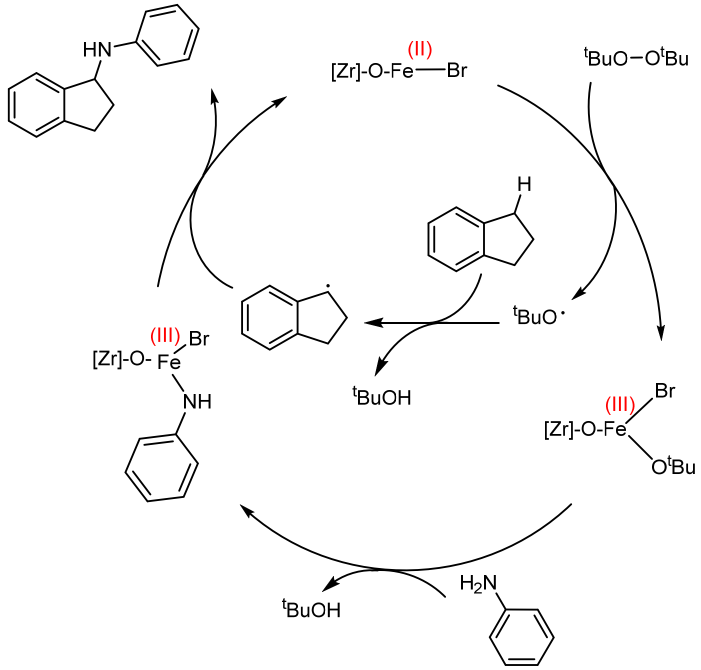

Supplementary Figure 44 / Proposed Fe(II)-Fe(III) cycle mechanism for UiO-FeBr catalyzed C–H amination.

Supplementary Tables

Supplementary Table 1 / Crystallographic information.

**Name**	**UiO-CoCl**	**$Zr_6O_4(OH)_4(McO)_{12}$**
**Formula**	$Zr_6O_4(OH)_4(C_{20}H_{12}O_4)_6$	$Zr_6O_4(OH)_4(C_4H_5O_2)_{12}\cdot 3(C_4H_6O_2)$
**Fw**	2577.24	1951.52
**Temperature (K)**	100	100
**Wavelength (Å)**	0.41328	0.71073
**Crystal system**	Cubic	Trigonal
**Space group**	$Fm\overline{3}m$	$P31c$
***a*, Å**	32.632(8)	17.2314(16)
***b*, Å**	32.632(8)	17.2314(16)
***c*, Å**	32.632(8)	18.0455(17)
**α, °**	90	90
**β, °**	90	90
**γ, °**	90	120
***V*, Å^3**	34748(26)	4640.2(10)
Z	4	2
**Density (calcd. g/cm^3)**	0.492	1.397
**Absorption coeff. (mm^{-1})**	0.271	0.724
**F(000)**	5152.0	1958.0
**θ range data collection**	0.628 – 12.345	2.257 – 21.620
**Limiting indices**	-33<=h<=33<br>-32<=k<=32<br>-33<=l<=33	-17<=h<=17<br>-17<=k<=15<br>-18<=l<=18
**Reflection collected**	42067	24114
**Independent reflections**	1071	3603
**R(int)**	0.3798	0.0413
**Data/restraints/parameters**	1071/66/48	3603/409/314
**Goodness-of-fit on F^2**	1.218	1.136
**Final R indices [I>2σ(I)]**	R1=0.0827, wR2=0.1725	R1=0.0636, wR2=0.1854
**R indices (all data)**	R1=0.1007, wR2=0.1828	R1=0.0785, wR2=0.1984

Supplementary Table 2 / Summary of EXAFS fitting parameters for Co-functionalized UiO-68 materials. Fits of the EXAFS region were performed using the Artemis program of the IFEFFIT package. Fits were performed with a *k*-weight of 2 in *R*-space. Refinement was performed by optimizing an amplitude factor S_0^2 and energy shift ΔE_0 which are common to all paths, in addition to parameters for bond length (ΔR) and Debye-Waller factor (σ^2). The fitting models were obtained from Material Studio 7.0, based on the crystal structure of UiO-68 or $Zr_6(OH)_4O_4(OMc)_{12}$ (reference). Unique parameters for ΔR and σ^2 were provided for all scattering paths in all fits.

**Sample**	**UiO-CoCl**	**UiO-CoH**	**UiO-Co-Bpin (HBpin)**	**UiO-Co-Bpin (B_2pin_2)**
**Fitting range**	*k* 2.80 – 11.70 $Å^{-1}$ *R* 1.0 – 5.0 Å	*k* 2.80 – 10.60 $Å^{-1}$ *R* 1.0 – 5.4 Å	*k* 2.80– 11.40 $Å^{-1}$ *R* 1.0 – 5.3 Å	*k* 2.80– 11.40 $Å^{-1}$ *R* 1.0 – 5.3 Å
**Independent points**	22	22	23	23
**Variables**	14	14	15	15
**Reduced chi-square**	28.5	63.2	46.2	32.7
**R-factor**	0.009	0.007	0.017	0.004
S_0^2	1.000	1.000	1.000	1.000
ΔE_0**(eV)**	-5.48±1.81	-3.60±2.68	-5.82±2.85	-4.17±1.22
R **(Co-O6) (Å)**	1.97±0.27	1.95±0.52	2.01±0.06	1.90±0.04
R **(Co-O7) (Å)**	1.98±0.14	1.96±0.29	1.95±0.06	1.99±0.02
R **(Co-C25) (Å)**	2.17±0.08	2.09±0.08	2.48±0.02	2.12±0.02
R **(Co-O22) (Å)**	2.62±0.04	2.99±0.06	2.80±0.05	2.86±0.03
R **(Co-Zr1) (Å)**	2.90±0.06	3.08±0.15	2.82±0.13	3.00±0.14
R **(Co-C26) (Å)**	3.36±0.04	3.52±0.12	3.12±0.05	3.10±0.04
R **(Co-O10) (Å)**	3.42±0.05	3.32±0.06	3.27±0.04	3.27±0.04
R **(Co-C28) (Å)**	3.45±0.05	3.35±0.06	3.30±0.04	3.30±0.04
R **(Co-C8) (Å)**	3.76±0.04	3.93±0.12	3.52±0.05	3.51±0.04
R **(Co-Zr2) (Å)**	3.77±0.06	3.63±0.08	3.67±0.04	3.63±0.04
R **(Co-C23) (Å)**	4.26±0.04	4.42±0.12	4.02±0.05	4.00±0.04
σ^2 **(Co-O) ($Å^2$)**	0.010±0.002	0.009±0.002	0.004±0.003	0.003±0.002
σ^2 **(Co-C) ($Å^2$)**	0.006±0.006	0.007±0.005	0.002±0.002	0.003±0.002
σ^2 **(Co-Zr) ($Å^2$)**	0.016±0.006	0.016±0.007	0.013±0.005	0.020±0.004
R **(Co-Cl) (Å)**	2.19±0.05	N.A.	N.A.	N.A.
σ^2 **(Co-Cl) ($Å^2$)**	0.009±0.008	N.A.	N.A.	N.A.
R **(Co-H) (Å)**	N.A.	1.43±0.11	N.A.	N.A.
σ^2 **(Co-H) ($Å^2$)**	N.A.	0.006±0.018	N.A.	N.A.
R **(Co-B) (Å)**	N.A.	N.A.	1.87±0.07	1.90±0.04
σ^2 **(Co-B) ($Å^2$)**	N.A.	N.A.	0.001±0.0003	0.001±0.0002
R **(Co-O(pin)) (Å)**	N.A.	N.A.	2.95±0.07	3.06±0.03

Supplementary Table 3 / Summary of EXAFS fitting parameters for $Zr_6(OCoCl)_4O_4(OMc)_{12}$.

**Sample**	$\mathbf{Zr_6(OCoCl)_4O_4(OMc)_{12}}$
**Fitting range**	*k* 3.00 – 11.85 $Å^{-1}$<br>*R* 1.18 – 4.98 Å
**Independent points**	21
**Variables**	14
**Reduced chi-square**	59.6
**R-factor**	0.002
S_0^2	1.000
ΔE_0**(eV)**	6.03±1.23
R **(Co-O6) (Å)**	2.05±0.37
R **(Co-O7) (Å)**	2.05±0.19
R **(Co-C25) (Å)**	2.34±0.05
R **(Co-O22) (Å)**	3.05±0.02
R **(Co-Zr1) (Å)**	3.10±0.04
R **(Co-C26) (Å)**	3.52±0.04
R **(Co-O10) (Å)**	3.33±0.02
R **(Co-C28) (Å)**	3.36±0.02
R **(Co-C8) (Å)**	3.92±0.04
R **(Co-Zr2) (Å)**	3.91±0.03
R **(Co-C23) (Å)**	4.41±0.04
σ^2 **(Co-O) (**$Å^2$**)**	0.006±0.001
σ^2 **(Co-C) (**$Å^2$**)**	0.010±0.005
σ^2 **(Co-Zr) (**$Å^2$**)**	0.013±0.002
R **(Co-Cl) (Å)**	2.31±0.01
σ^2 **(Co-Cl) (**$Å^2$**)**	0.002±0.001

Supplementary Table 4 / Summary of EXAFS fitting parameters for UiO-FeBr.

**Sample**	**UiO-FeBr**
**Fitting range**	*k* 3.00 – 11.80 $Å^{-1}$<br>*R* 1 – 5.1 Å
**Independent points**	23
**Variables**	15
**Reduced chi-square**	81.7
**R-factor**	0.012
S_0^2	1.000
ΔE_0**(eV)**	-2.99±1.90
R **(Co-O6) (Å)**	1.98±0.05
R **(Co-O7) (Å)**	2.00±0.03
R **(Co-C25) (Å)**	2.44±0.0004
R **(Co-O22) (Å)**	2.58±0.05
R **(Co-Zr1) (Å)**	2.84±0.05
R **(Co-C26) (Å)**	3.45±0.08
R **(Co-O10) (Å)**	3.48±0.08
R **(Co-C28) (Å)**	3.51±0.08
R **(Co-C8) (Å)**	3.85±0.08
R **(Co-Zr2) (Å)**	3.59±0.09
R **(Co-C23) (Å)**	4.34±0.08
σ^2 **(Co-O)** ($Å^2$)	0.011±0.001
σ^2 **(Co-C)** ($Å^2$)	0.016±0.019
σ^2 **(Co-Zr)** ($Å^2$)	0.018±0.004
R **(Co-Cl) (Å)**	2.41±0.05
σ^2 **(Co-Cl)** ($Å^2$)	0.015±0.004

Supplementary Table 5 / Optimization of benzylic C–H borylation of methylarenes catalyzed by UiO-Co materials. Synthesis and characterization of UiO-66-CoCl and UiO-67-CoCl. UiO-66-CoCl and UiO-67-CoCl were prepared by post-synthetic metalation with $CoCl_2$, using the same method for synthesizing UiO-CoCl described above. UiO-66-CoCl and UiO-67-CoCl have 100% Co-loading with respect to μ^3-OH centers based on ICP-MS analysis.

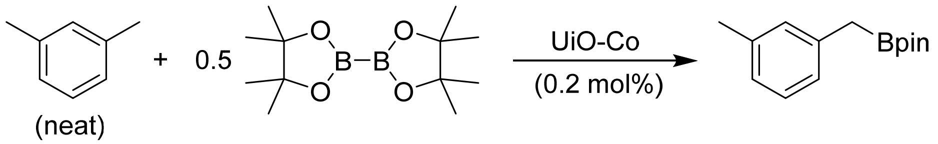

Entry	Catalyst (0.2 mol% Co)	Temperature (°C)	Time (d)	% Conversion [Benzyl:Ar]
1	UiO-66-Co	103	2.5 d	21 (55:45)
2	UiO-67-Co	103	2.5 d	25 (40:60)
**3**	**UiO-Co**	**103**	**2.5 d**	**100 (96:4)**
4	UiO-Co	106	2.5 d	100 (88:12)
5	UiO-Co	96	3 d	100 (96:4)
6	UiO-Co	110	2.5 d	<50

[a]Reaction conditions: 1.0 mg of UiO-CoCl, 5 equiv $NaBEt_3H$ (1.0 M in THF), *m*-xylene (2 mL), B_2pin_2, N_2.

Supplementary Table 6: The retention time of GC traces.

Compounds	Retention time (min)	Compounds	Retention time (min)
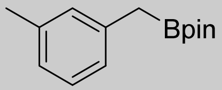	41.7	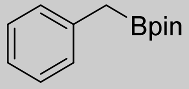	36.9
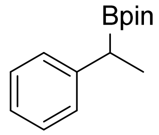	37.2	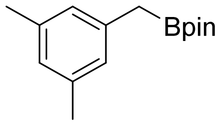	46.2
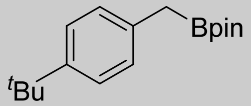	51.8	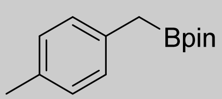	42.6
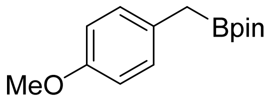	53.4		
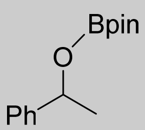	25.3	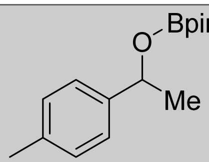	30.0

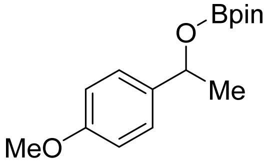	40.7	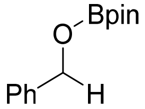	25.3

Supplementary Table 7 / C–H borylation of methylarenes catalyzed by Co-functionalized homogeneous SBU $Zr_6(OCoCl)_4O_4(OMc)_{12}$. In the homogeneous cluster, the observed lower selectivity for benzylic C-H can be explained by the relatively smaller steric hindrance produced by the methylethylenyl group than the phenyl group.

Entry	Substrate	Product(s)	Precatalyst (mol % Co)	Time	% Yield [Benzyl:Ar]
**1**	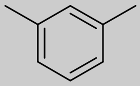	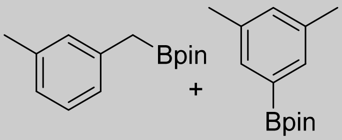	UiO-CoCl (0.2)	2.5 d	92 [96:4]
			$Zr_6O_4(OCoCl)_4(McO)_{12}$ (2.0)	5 d	82 [67:33]
**2**	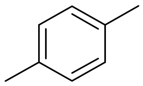	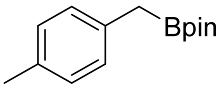	$Zr_6O_4(OCoCl)_4(McO)_{12}$ (2.0)	5 d	56

Supplementary Table 8 / Dependence of catalytic activity on benzylic C–H borylation catalytic activity on substrate size.[a]

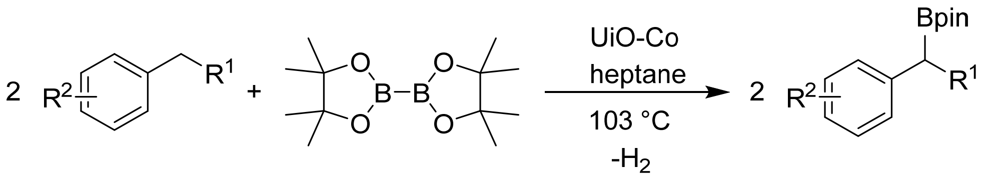

Entry	Substrate	Product(s)	% Co-loading	Time	% Yield
**1**	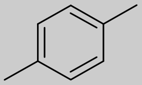	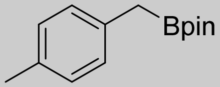	0.2	2.5 d	94
**2**	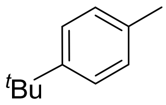	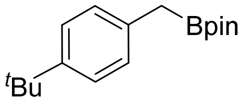	0.2	2.5 d	22
**3**	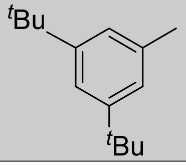	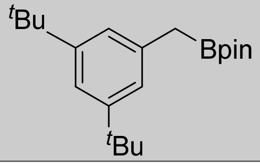	0.2	2.5 d	6

[a]Reaction conditions: 1.0 mg of UiO-CoCl, 5 equiv $NaBEt_3H$ (1.0 M in THF), arene, B_2pin_2, 103 °C, heptanes (2 mL), reflux under N_2.

Supplementary Table 9 / C–H silylation of toluene catalyzed by Co-functionalized homogeneous SBU $Zr_6(OCoCl)_4O_4(OMc)_{12}$ and UiO-CoCl under identical reaction conditions shows activity of UiO-Co is higher than the homogeneous SBU analog.

**Entry**	**Substrate**	**Product(s)**	**Precatalyst (mol % Co)**	**Time**	**% Yield [Benzyl:Ar]**
1	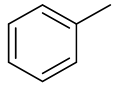	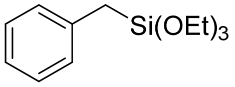	UiO-CoCl (0.4)	3 d	85
2			$Zr_6O_4(OCoCl)_4(McO)_{12}$ (0.4)	3 d	0

*Reaction conditions: 0.4 mol % Co, 3 equiv of $NaBEt_3H$ (1.0 M in THF) w.r.t. Co, toluene (1 mL), $(OEt)_3SiH$, 100 °C.

Supplementary Table 10 / Hydrogenation of 1-octene catalyzed by Co-functionalized homogeneous SBU $Zr_6(OCoCl)_4O_4(OMc)_{12}$ and UiO-CoCl under identical reaction conditions shows activity of UiO-Co is higher than the homogeneous SBU analog.

**Entry**	**Substrate**	**Product(s)**	**Precatalyst (mol % Co)**	**Time**	**% Yield**
1	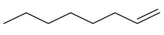	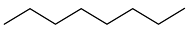	UiO-CoCl (0.002)	1 h	100
2			$Zr_6O_4(OCoCl)_4(McO)_{12}$ (0.002)	1 h	11

*Reaction conditions: 0.002 mol % Co, 3 equiv of $NaBEt_3H$ (1.0 M in THF) w.r.t. Co, THF (1 mL), 1-octene, H_2 (40 bar), 23 °C.

Supplementary Table 11 / Hydroboration of styrene catalyzed by Co-functionalized homogeneous SBU $Zr_6(OCoCl)_4O_4(OMc)_{12}$ and UiO-CoCl under identical reaction conditions shows that the activity of UiO-Co is higher than the homogeneous SBU analog.

**Entry**	**Substrate**	**Product(s)**	**Precatalyst (mol % Co)**	**Time**	**% Yield**
1	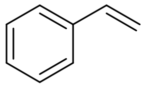	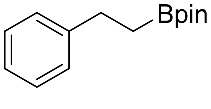	UiO-CoCl (0.1)	3d	93
2	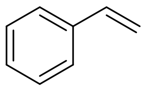	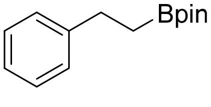	$Zr_6O_4(OCoCl)_4(McO)_{12}$ (0.1)	3d	26

*Reaction conditions: 0.1 mol % Co, 3 equiv of $NaBEt_3H$ (1.0 M in THF) w.r.t. Co, THF (1 mL), styrene, HBpin, 70 °C.

Supplementary Table 12 / C-H amination of indane catalyzed by Fe-functionalized homogeneous SBU $Zr_6(OFeBr)_4O_4(OMc)_{12}$ and UiO-FeBr under identical reaction conditions shows that the activity of UiO-FeBr is higher than the homogeneous SBU analog.

**Entry**	**Substrate**	**Product**	**Precatalyst (mol % Fe)**	**Time**	**% Yield**
1	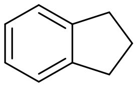	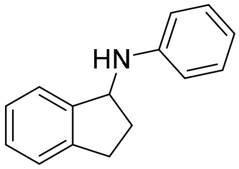	UiO-FeBr (10)	3 d	53%
2	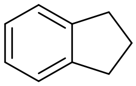	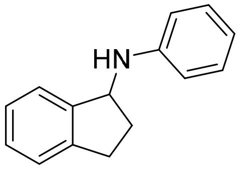	$Zr_6O_4(OFeBr)_4(McO)_{12}$ (10)	3 d	33%

*Reaction conditions: 8 μmol of MOF-FeBr w.r.t. Fe, indane (3.2 mmol, neat), aniline (0.16 mmol), $(^t\text{BuO})_2$ (0.48 mmol, 3.0 eq), 100 °C.

Supplementary Discussion

Experiments to confirm that carboxylates of UiO-MOF were remained intact in presence of nBuLi before metalation with $CoCl_2$.

(a) Digestion procedure for lithiated UiO-68. 5 mg of UiO-68 in THF was treated with 10 equiv. of ^nBuLi (2.5 M in Hexanes) and stirred slowly for 6 hours. Then the lithiated UiO-68 was washed sequencially with THF three times to remove excess ^nBuLi, then three times with benzene to remove THF, and dried under vacuum for two hours. The dry lithiated UiO-68 was treated with 1 mL of 5% D_2SO_4 in DMSO-d_6 and sonificated for 10 min to give clear colorless solution. This step breaks the coordination bond within the MOF to release all the organic ligands. The solution was directly used for ^1H NMR. The ^1H NMR spectra showed the presence of only 1,4-bis(4-carboxyphenyl)benzene (Supplementary Figure 8).

(b) IR spectra of UiO-68 and UiO-68-CoCl. IR spectra of UiO-68-CoCl shows the presence of two strong carbonyl stretching frequencies at 1605 cm^{-1} and 1591 cm^{-1} similar to UiO-68 (Supplementary Figure 9a).

Test of "heterogeneity" of the MOF catalysis in alkyl C–H borylation.

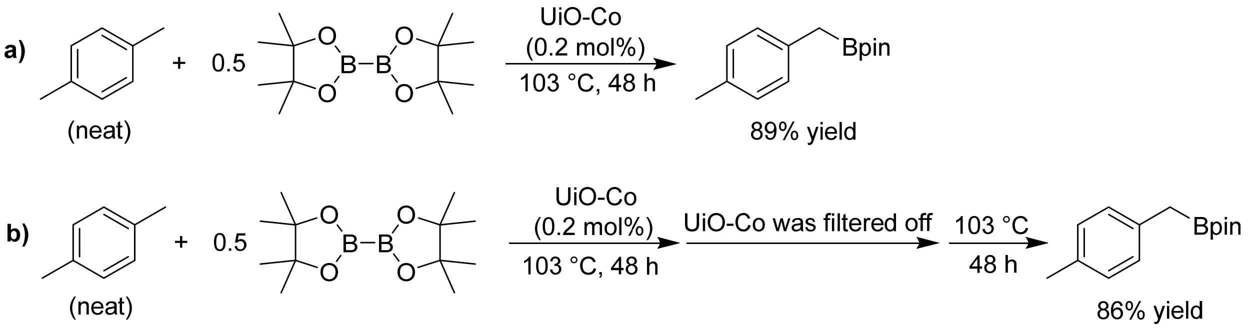

In a glovebox, UiO-CoCl (1.0 mg, 0.2 mol % Co) was charged into a small vial and 0.5 mL THF was added. Then, 15 μL $NaBEt_3H$ (1.0 M in THF) was added to the vial and the mixture was stirred slowly for 1 h in the glovebox. The solid was centrifuged out of suspension and washed twice with THF and then once with *p*-xylene. B_2pin_2 (43.0 mg, 0.169 mmol) in 2.0 mL *p*-xylene was added to the vial and the resultant mixture was transferred to a Schlenk tube. The tube was heated under nitrogen at 103 °C for 48 h to obtain the alkyl boronate ester in 89% yield as determined by GC analysis.

In a glovebox, UiO-CoCl (1.0 mg, 0.2 mol % Co) was charged into a small vial and 0.5 mL THF was added. Then, 15 μL $NaBEt_3H$ (1.0 M in THF) was added to the vial and the mixture was stirred slowly for 1 h in the glovebox. The solid was centrifuged out of suspension and washed twice with THF and then once with *p*-xylene. B_2pin_2 (43.0 mg, 0.169 mmol) in 2.0 mL *p*-xylene was added to the vial and the resultant mixture was transferred to a Schlenk tube. The tube was heated under nitrogen at 103 °C for 48 h. The solid catalyst was separated via centrifugation and the supernatant was filtered through a Celite. Then, the supernatant was stirred at 103 °C for an additional 48 h. GC

analysis showed that the alkyl-boronate ester was obtained in 86% yield. These two reactions afforded almost the same yields. This experiment shows that the UiO-Co is the actual catalyst for benzylic C–H borylation.

Control experiments with trapped Co-species within UiO-68.

(a) The effects of any trapped Co-species. We performed control experiments with UiO-68. UiO-68 (12 mg) was transferred to a vial containing 5 mL THF solution of $CoCl_2$ (4.0 mg). The mixture was stirred slowly for 16 h, and the solid was then centrifuged out and washed with THF 5-8 times over 24 h. The white color of UiO-68 remained unchanged upon metalation. A small amount of Co was detected by ICP-MS (Co:Zr = 0.018:1; equivalent to 2.7% Co loading based on the lithiation approach). Upon treatment of $NaEt_3BH$, the resulting solid was found to be inactive for benzylic C–H borylation of neat *p*-xylene at 103 °C (0.2 mol % Co loading). These observations thus proved that the surface trapped nanoparticles contribute little, if any, to the catalytic activity of the MOF catalyst.

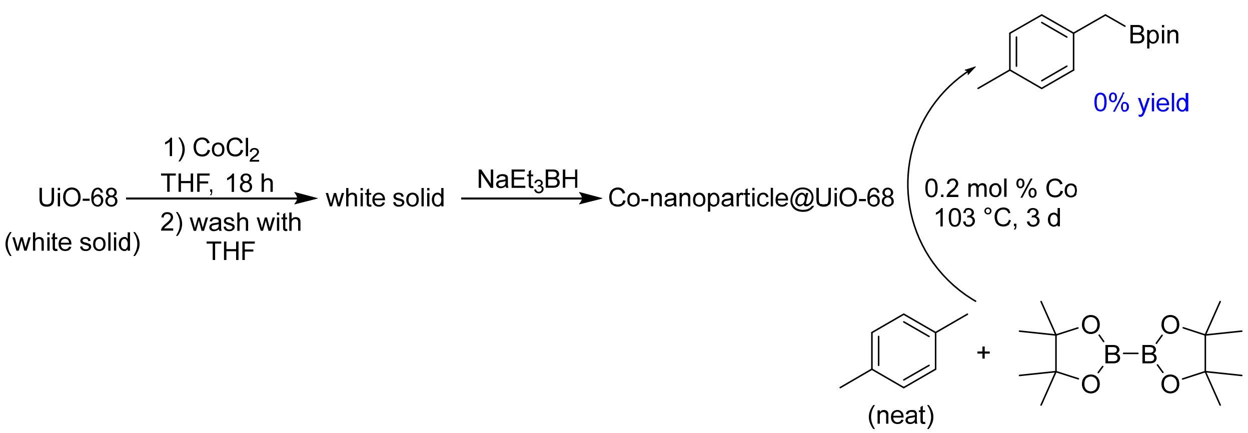

(b) Control experiment with Co-nanoparticles. To a THF solution (0.2 mL) of $CoCl_2$ (1.0 mg), $NaEt_3BH$ (23 μL) was added. A black precipitate formed immediately. The resulting mixture was stirred for 1 h and then transferred to a Schenk tube containing B_2pin_2 (19.6 mg, 0.077 mmol) dissolved in 1.5 mL of *p*-xylene. The resultant mixture was heated under N_2 at 103 °C for 2 days. The GC analysis showed no formation of boronate ester.

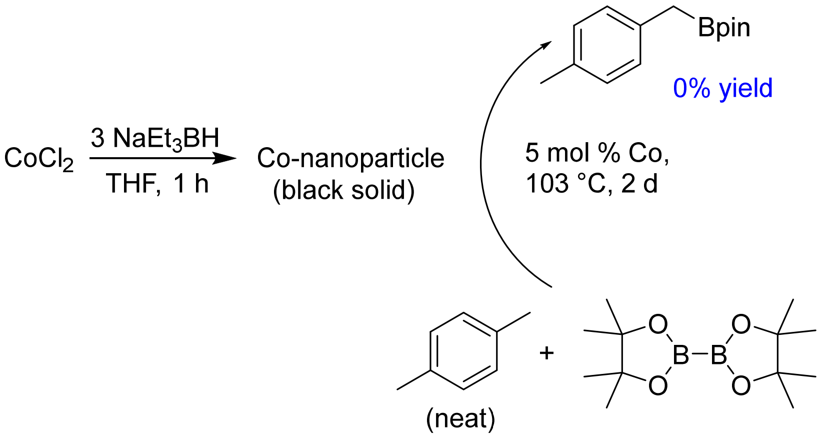

Investigation of substrate size effect on catalytic activity in benzylic C–H borylation.

In a glovebox, UiO-CoCl (1.0 mg, 0.2 mol % Co) was charged into a small vial and 0.5 mL THF was added. Then, 15 µL $NaBEt_3H$ (1.0 M in THF) was added to the vial and the mixture was stirred slowly for 1 h in the glovebox. The solid was centrifuged out of suspension and washed with THF twice and then once with heptane. B_2pin_2 (43.0 mg, 0.169 mmol) and *p*-xylene (41.8 µL, 0.34 mmol) in 2.0 mL heptane was added to the vial and the resultant mixture was transferred to a Schlenk tube. The tube was heated under nitrogen at 103 °C for 2.5 d to obtain the alkyl boronate ester in 94% yield as determined by GC analysis.

The borylation reaction of 4-tert-butyl-toluene and 3,5-di-tert-butyl-toluene were conducted using the same procedure described above under identical reaction conditions and the results are summarized in Supplementary Table 7. The yield of the boronate ester decreased dramatically on increasing the size of the substrates. Therefore, this experiment demonstrates that catalysis is facilitated by Co-sites both inside the pores and on the outside of the MOFs, not the framework surface alone.

Test of "heterogeneity" of the MOF catalysis in alkene hydrogenation.

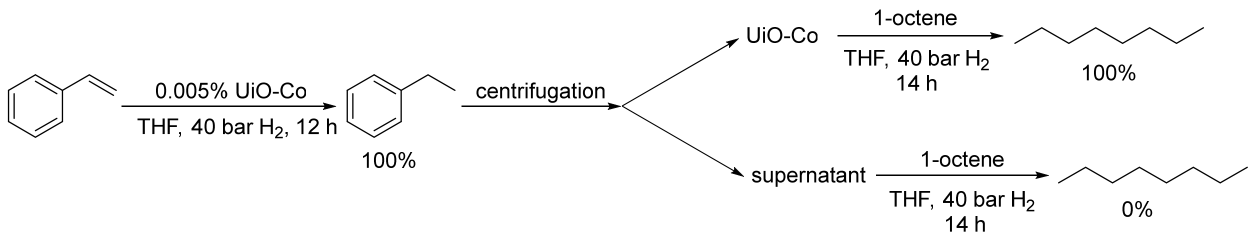

In a nitrogen-filled glove box, UiO-CoCl (0.5 mg, 0.005 mol % Co) in 1.0 mL THF was charged into a glass vial. $NaBEt_3H$ (15 µL, 1.0 M in THF) was then added to the vial and the mixture was stirred for 1 hour. The solid was then centrifuged, washed with THF twice, and transferred to a glass vial containing 0.5 mL THF. Styrene (0.71 g, 6.82 mmol) was then added to the vial. The vial was then placed in a Parr reactor which was sealed under nitrogen atmosphere and later charged with hydrogen to 40 bar. After 12 h, the pressure was released and the MOF catalyst was centrifuged out from suspension. Styrene was completely converted to ethylbenzene as determined by ^1H NMR spectra based on the integration of substrate and product peaks in the crude.

After the solid and supernatant were separated, 1-octene (1.07 mL, 6.82 mmol) was added to each of the portions which were later placed in a Parr reactor, sealed under nitrogen and charged with hydrogen to 40 bar. After 14 h, the pressure was released and the supernatant was separated from the solid catalyst when necessary. Conversions of 1-octene to *n*-octane determined based on integration of substrate and product peaks in the crude ^1H NMR spectra were 100% in the presence of UiO-Co and 0% in the presence of the supernatant. This experiment shows that UiO-Co is the actual catalyst for alkene hydrogenation.

Supplementary Methods

General Experimental. All of the solvents were purchased from Fisher and used without further purification unless otherwise noted. All of the other substrates and reagents are commercially available and used as received unless otherwise indicated. 1-octene, styrene, α-methylstyrene, *cis*-β-methylstyrene, allyl acetate, and benzaldehyde were distilled and then dried over freshly activated 4 Å molecular sieves prior to use. Cyclohexene, *m*-xylene, *p*-xylene, ethylbenzene, 4-tert-butyltoluene, 4-methylanisole, cumene, *p*-cymene, acetophenone and 2-acetylthiophene were purchased from Fisher and were degassed and then dried with freshly activated 4 Å molecular sieves in a glovebox prior to use. Pinacolborane and aniline were purchased from Fisher and was freshly distilled prior to use. Et_3SiH and $(EtO)_3SiH$ were purchased from Fisher and were used as received in the glovebox. ^1H NMR spectra were recorded on a Bruker NMR 400 DRX spectrometer at 400 MHz and referenced to the proton resonance resulting from incomplete deuteration of the deuterated chloroform (δ 7.26) or deuterated DMSO (δ 2.50). Thermogravimetric analysis (TGA) was performed in air using a Shimadzu TGA-50 equipped with a platinum pan. Powder X-ray diffraction (PXRD) patterns were collected on a Bruker D8 Venture, dual microsource (Cu and Mo) diffractometer with a CMOS detector. Cu Kα radiation was used. The PXRD patterns were processed with the APEX 2 package using PILOT plug-in. Background diffraction signal from glass capillary tube and solvent at 2θ~20º was simulated and removed by the program PowderX. ICP-MS data were obtained with an Agilent 7700x ICP-MS and analyzed using ICP-MS MassHunter version B01.03. Samples were diluted in a 2% HNO_3 matrix and analyzed with a ^{159}Tb internal standard against a six-point standard curve over the range from 0.1 ppb to 1000 ppb. Mass spectra were obtained with an Agilent 6224 T (HRA-MS) Mass Spectrometer. The correlation coefficient was >0.9997 for all analytes of interest. Data collection was performed in Spectrum Mode with five replicates per sample and 100 sweeps per replicate.

Ligand synthesis

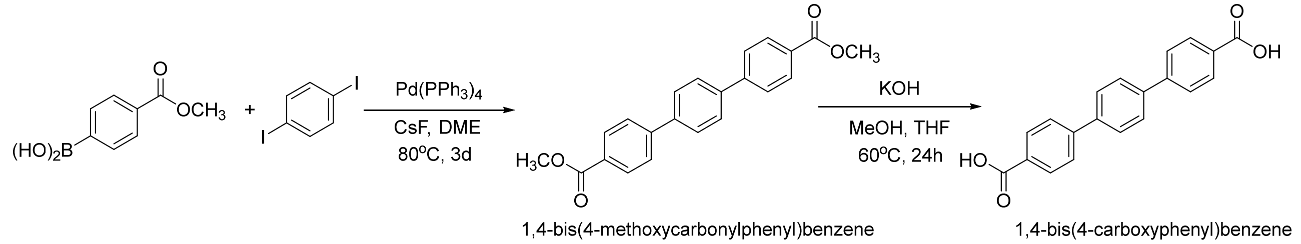

1,4-bis(4-methoxycarbonylphenyl)benzene (CAS: 15493-26-2): 1,4-diiodobenzene (1.00 g, 3.03 mmol) and 4-methoxycarbonylphenylboronic acid (1.64 g, 9.09 mmol) were suspended in 94 mL of 1,2- dimethoxyethane in a glove box. Tetrakis(triphenylphosphine) palladium (175 mg, 0.152 mmol) and cesium fluoride (4.14 g, 27.3 mmol) were then added. The resulting mixture was sealed in a pressure vessel under nitrogen and stirred at 80 ºC for 3 days. After cooling to room temperature, the reaction mixture was mixed with H_2O (20 mL), and centrifuged to obtain solid crude compound. The solid was then washed sequentially with H_2O, dimethoxylethane, and THF to remove

impurities and dried in vacuo to afford 1,4-bis(4-methoxycarbonylphenyl)benzene as a white solid (420 mg, 1.21 mmol, 40% yield). ^{1}H NMR (500 MHz, $CDCl_3$): δ 8.14 (d, 2 H, $^3J_{HH}$ = 8.4 Hz), 7.74 (s, 4 H), 7.72 (d, 4 H, $^3J_{HH}$ = 8.4 Hz), 3.96 (s, 6 H). HRMS (ESI-TOF, *m/z*), calcd for $C_{22}H_{18}O_4$ $[M+Na]^+$ 369. 1103, found 369.1079.

1,4-bis(4-carboxyphenyl)benzene[1] (CAS: 13653-84-4): 1,4-bis(4-carboxyphenyl)benzene (420 mg, 1.21 mmol) was suspended in THF (65 mL). A solution of KOH (6.17 g, 110 mmol) dissolved in MeOH (20 mL) was then added, and the reaction mixture was stirred at 60 ºC for 24 h. The suspension was cooled to room temperature and the resulting precipitate was collected by centrifugation. The solution was washed with dry THF (20 mL) and recollected by centrifugation. The solid was suspended in THF (20 mL) and trifluoroacetic acid (3 mL) was slowly added and stirred for 1.5h at room temperature. H_2O (15 mL) was then added, and the white solid was isolated by centrifugation, washed with THF and Et_2O, and dried in vacuo to obtain 1,4-bis(4-carboxyphenyl)benzene (331.9 mg, 1.04 mmol, 86% yield) as pale-white solid. ^{1}H NMR (500 MHz, DMSO-d_6) δ 13.00 (br s, 2 H), 8.05 (d, 4 H, $^3J_{HH}$ = 8.3 Hz), 7.89 (s, 4H), 7.88 (d, 4 H, $^3J_{HH}$ = 8.3Hz) (Supplementary Figure 1).

Synthesis and characterization of the UiO-68 MOF[2]

$ZrCl_4$ (1.30 mg, 5.03 μmol), 1,4-bis(4-carboxyphenyl)benzene (1.6 mg, 5.53 μmol) were dissolved in 0.8 mL of DMF in 1 dram vial, and 15.4 μL of trifluoroacetic acid was then added. The vial was capped and then heated at 120 ºC for 3 days to afford a white solid as the MOF product (2.0 mg, 95% yield). Comparing powder X-ray diffraction pattern of obtained MOF with simulated pattern from single crystal structure shows good crystallinity of the UiO-68-MOF (Supplementary Figure 2 and 3).

Synthesis and characterization of UiO-CoCl. In a glovebox, UiO-68 (20.0 mg) in 3 mL THF was cooled to -30 °C for 30 min. To the cold suspension, 33 μL of *n*BuLi (2.5 M in hexanes) was added dropwise and the resultant light yellow mixture was stirred slowly overnight at room temperature. The light yellow solid was centrifuged out and washed with THF 5-6 times over 6 h. Then, the lithiated UiO-68 was transferred to a vial containing 5 mL THF solution of $CoCl_2$ (6.0 mg). The mixture was stirred for 15 h and the deep blue solid was then centrifuged out and washed with THF for 5-8 times. The metalated MOFs were then stored in THF in the glovebox for further uses. UiO-CoCl has 45% solvent weight based on TGA analysis (Supplementary Figure 5), and 100% Co-loading with respect to μ_3-OH centers based on ICP-MS analysis.

Synthesis and characterization of UiO-FeBr. In a glovebox, UiO-68 (20.0 mg) in 3 mL THF was cooled to -30 °C for 30 min. To the cold suspension, 33 μL of *n*BuLi (2.5 M in hexane) was added dropwise and the resultant light yellow mixture was stirred slowly overnight at room temperature. The light yellow solid was centrifuged out and washed with THF 5-6 times over 6 h. Then, the lithiated UiO-68 was transferred to a vial containing 5 mL THF

solution of $FeBr_2(THF)_2$ (16.5 mg). The mixture was stirred for 15 h before the dark brown solid was centrifuged out and washed with THF 5-8 times. The metalated MOFs were then stored in THF in the glovebox for further uses. UiO-FeBr has 44% solvent weight based on TGA analysis (Supplementary Figure 13) and 100% Fe-loading with respect to μ^3-OH centers based on ICP-MS analysis.

Synthesis of homogeneous Zr_6 cluster and its metalation with $CoCl_2$

Synthesis of $Zr_6O_4(OH)_4(McO)_{12}$ cluster: The homogeneous cluster $Zr_6O_4(OH)_4(McO)_{12}$ analogous to SBUs of UiO-68 was synthesized using a method modified from the literature.[3] To a 20 mL vial, $Zr(O^nPr)_4/^nPrOH$ (2 mL, 6.2 mmol) and methyl acrylic acid (2 mL, 23.6 mmol) were added and mixed through gentle shaking. The mixture was left undisturbed for five days. Column-shaped colorless crystals were observed and isolated via filtration (417.6 mg, 24% yield). The structure of the compound was solved via single crystal diffraction (Supplementary Figure 18, Supplementary Table 1).

Metalation of the homogeneous Zr_6-cluster with $CoCl_2$

Synthesis of $Zr_6O_4(OCoCl)_4(McO)_{12}$. $Zr_6O_4(OH)_4(McO)_{12}$ (158 mg, 372 μmol of μ_3-OH group) was dissolved in THF (2 mL). After cooling to -30 ºC in a freezer in a glove box, *n*BuLi (150 mL, 2.5 M in THF, 1 equiv.) was added and stirred for 1 h. $CoCl_2$ (48.3 mg, 372 μmol, 1 equiv.) was subsequently added and stirred overnight, then dried under vacuum to remove the THF. Et_2O was added to the obtained blue solid to precipitate LiCl followed by filtration through Celite. The filtrate was concentrated under vacuum and recrystallized using Et_2O/pentane, and then a blue powdery solid (92 mg, 177 mmol of μ^3O-CoCl, 48% yield) was obtained. ICP-MS analysis of the digested sample give Zr:Co ratio of 1.33±0.04, which is close to the expected ratio of 1.50. HRMS (ESI-TOF, Positive Charge), m/Z calcd for $C_{48}H_{60}Cl_4Co_4O_{32}Zr_6$ $[M+2Na]^{2+}$: 1059.91; Found: 1059.69.

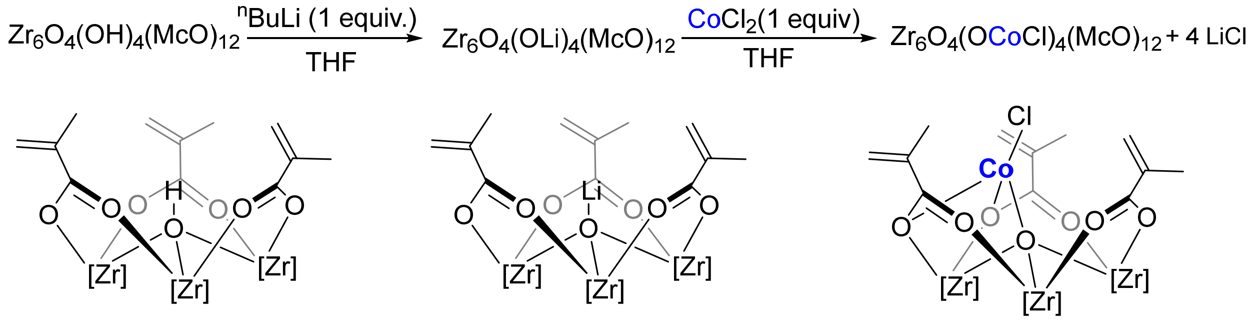

Crystallographic Information and Structural Figures.

Single crystal X-ray diffraction of UiO-CoCl was performed with a Bruker APEX II CCD-based detector at ChemMatCARS (Sector 15), Advanced Photon Source (APS), Argonne National Laboratory. Single crystal X-ray diffraction of $Zr_6O_4(OH)_4(McO)_{12}$ was performed with a Bruker D8 Venture, dual microsource (Cu and Mo)

diffractometer with the PHOTON 100 CMOS detector. Mo Kα radiation was used. Data reduction and integration were performed with the Bruker APEX3 software package (Bruker AXS, version 2015.5-2, 2015). Data were scaled and corrected for absorption effects using the multi-scan procedure as implemented in SADABS (Bruker AXS, version 2014/5, 2015, part of Bruker APEX3 software package). The structure was solved by SHELXT (Version 2014/5) [4] and refined by a full-matrix least-squares procedure using OLEX2[5] software packages (XL refinement program version 2014/7)[6]. Crystallographic data and details of the data collection and structure refinement are listed in Supplementary Table 1.

X-Ray Absorption Spectroscopic Analysis

X-ray absorption data were collected at Beamline 9-BM-C at the Advanced Photon Source (APS) at Argonne National Laboratory. Spectra were collected at the iron or cobalt K-edge in transmission mode. The X-ray beam was monochromatized by a Si(111) monochromater and detuned by 25% to minimize harmonics. A metallic iron or cobalt foil standard was used as the reference for energy calibration and was measured simultaneously with experimental samples. The incident beam intensity (I_0) was measured by an ionization chamber with 30% N_2 and 70% He gas composition. Data was collected in three regions: a pre-edge region –150 to –20 eV (5 eV step size, dwell time 1.0 s), XANES region – 20 to 50 eV (0.5 eV step size, dwell time 1.0 s), and EXAFS region 3.62 Å $^{-1}$ to 13.93 Å $^{-1}$ (0.05 Å $^{-1}$ step size, dwell time increased linearly from 1.0 to 3.9 seconds over the region to facilitate higher k-weighted data processing). All energies are listed relative to the elemental Fe K-edge (7112 eV) of Co K-edge (7709 eV). Multiple X-ray absorption spectra were collected at room temperature for each sample. Samples were ground and mixed with polyethyleneglycol (PEG) and packed into a 6-shooter sample holder to achieve adequate absorption length.

Data were processed using the Athena and Artemis programs of the IFEFFIT package based on FEFF 6.[7,8] Prior to merging, spectra were calibrated against the reference spectra (metallic Co or Fe) and aligned to the first peak in the smoothed first derivative of the absorption spectrum, background removed, and spectra processed to obtain a normalized unit edge step.

Procedures for Catalytic Benzylic C–H Borylation

A typical procedure for UiO-Co catalyzed benzylic C–H borylation of arenes.

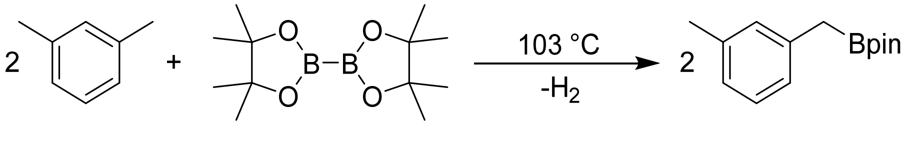

In a glovebox, UiO-CoCl (1.0 mg, 0.2 mol % Co) was charged into a small vial and 0.5 mL THF was added. Then, 15 μL $NaBEt_3H$ (1.0 M in THF) was added to the vial and the mixture was stirred slowly for 1 h in the glovebox. The solid was centrifuged out of suspension and washed with THF twice and then once with *m*-xylene. B_2pin_2 (43.0 mg, 0.169 mmol) in 2.0 mL *m*-xylene was added to the vial and the resultant mixture was transferred to a Schlenk

tube. The tube was heated under nitrogen at 103 °C for 3 d. The reaction mixture was cooled to room temperature and the solid was centrifuged out of suspension. The extract was passed through a short plug of celite and then concentrated *in vacuo* to give boronate ester product in 92% yield (72 mg, 0.311 mmol). The crude product was a mixture of alkyl borate ester (2-(3-methylbenzyl)-4,4,5,5-tetramethyl-1,3,2-dioxaborolane) and arene boronate ester (5-(4,4,5,5-tetramethyl-1,3,2-dioxaborolan-2-yl)-*m*-xylene) in 96:1 ratio as determined by GC analysis. ^{1}H NMR of 2-(3-methylbenzyl)-4,4,5,5-tetramethyl-1,3,2-dioxaborolane ($CDCl_3$, 400 MHz): δ 7.12 (t, $^{3}J_{HH}$ = 7.5 Hz, 1 H), 7.00 (s, 1 H), 7.00 (s, 1 H), 6.94 (d, $^{3}J_{HH}$ = 7.6 Hz, 1 H), 2.31 (s, 3 H), 2.26 (s, 2 H), 1.24 (s, 12 H). ^{11}B NMR (128 MHz, CDCl3): δ 33.1. The ^{1}H and ^{11}B NMR data are similar to the reported data.[9]

Quantification of hydrogen production in the reaction of UiO-CoH with HBpin.

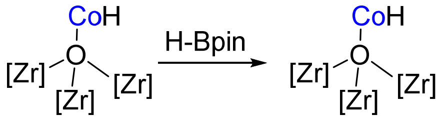

In a J. Young NMR tube, 2.25 mg of UiO-CoH in heptane was added. Then, HBpin (1.2 equiv. w.r.t. Co) was added to the mixture and then the tube was sealed quickly. The tube was left for 4 h. The headspace gas (total volume 1.5 mL) was analyzed by gas chromatography to give a hydrogen content of 2.8157% (v/v). The total amount of hydrogen in the headspace was then calculated to be:

$$1.5\ \text{mL} \times \frac{2.81577}{50} \times 101\ \text{kPa} \div 8.314\ \text{J}\cdot\text{mol}^{-1}\cdot\text{K}^{-1} \div 295\ \text{K} = 3.48\ \mu\text{mol}$$

The amount of hydrogen expected from the reaction of UiO-68-CoH and HBpin is 3.85 μmol, which is close to the experimental value.

Determination of the rate law for UiO-Co-catalyzed benzylic C–H borylation of *p*-xylene.

The rate law of the benzylic C–H borylation of *p*-xylene was determined by the method of initial rates (up to 10% conversion). The reactions were conducted in heptane (total volume of solution was 2.0 mL) in a Schlenk tube at 103 °C. The Schlenk tube was connected to a reflux condenser under N_2. To determine the rate dependence on one reagent, the concentration of that reagent was varied, while the concentration of other reagents and the total volume of the solution (2.0 mL) were held constant. After borylation reaction for 12 h, mesitylene (10 μL) was added to the reaction mixture and then the concentration of *p*-xylene was determined by GC using mesitylene as the internal standard. The rates refer to the rates of decrease of *p*-xylene in units of $M\cdot s^{-1}$. To determine the rate dependence on *p*-xylene, the concentration of *p*-xylene was varied between 0.8×10^{-1} – 2.03×10^{-1} M, while the concentration of Co was 2.71×10^{-4} M and concentration of B_2pin_2 was 8.5×10^{-2} M. To determine the rate dependence on catalyst, the concentration of Co was varied between 2.71×10^{-4} – 6.78×10^{-4} M, while the initial concentrations of *p*-xylene and

B_2pin_2 were 3.41×10^{-1} M and 1.70×10^{-1} M, respectively. To determine the rate dependence on B_2pin_2, the concentration of B_2pin_2 was varied between 3.94×10^{-2} – 9.84×10^{-2} M, while the concentrations of *p*-xylene and Co were 3.41×10^{-1} M and 2.0×10^{-4} M, respectively (Supplementary Figure 33).

2-(Benzyl)-4,4,5,5-tetramethyl-1,3,2-dioxaborolane.

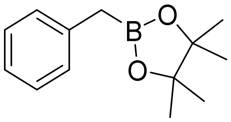

2-(Benzyl)-4,4,5,5-tetramethyl-1,3,2-dioxaborolane was prepared in 72% yield according to the general procedure using UiO-Co (1.0 mg, 0.2 mol % Co), B_2pin_2 (43.0 mg, 0.169 mmol) in toluene (2.0 mL). ^{1}H NMR (400 MHz, $CDCl_3$): δ 7.23 (d, $^3J_{HH}$ = 7.2 Hz, 2 H), 7.18 (d, $^3J_{HH}$ = 6.7 Hz, 2 H), 7.12 (t, $^3J_{HH}$ = 7.1 Hz, 1 H), 2.29 (s, 2 H), 1.23 (s, 12 H). ^{11}B NMR (128 MHz, $CDCl_3$): δ 32.5. The ^{1}H and ^{11}B NMR data are similar to the reported data.[9]

2-(3,5-Dimethylbenzyl)-4,4,5,5-tetramethyl-1,3,2-dioxaborolane.

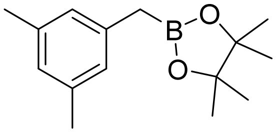

2-(3,5-Dimethylbenzyl)-4,4,5,5-tetramethyl-1,3,2-dioxaborolane was prepared in 94% yield according to the general procedure using UiO-Co (1.0 mg, 0.2 mol % Co), B_2pin_2 (43.0 mg, 0.169 mmol) in mesitylene (2.0 mL). ^{1}H NMR (400 MHz, $CDCl_3$): δ 6.81 (s, 2 H), 6.77 (s, 1 H), 2.27 (s, 6 H), 2.22 (s, 2 H), 1.24 (s, 12 H). ^{11}B NMR (128 MHz, CDCl3): δ 33.1. The ^{1}H and ^{11}B NMR data are similar to the reported data.[10]

2-(4-Methoxybenzyl)-4,4,5,5-tetramethyl-1,3,2-dioxaborolane.

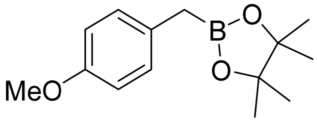

2-(4-Methoxybenzyl)-4,4,5,5-tetramethyl-1,3,2-dioxaborolane was prepared in 83% yield according to the general procedure using UiO-Co (1.0 mg, 0.2 mol % Co), B_2pin_2 (43.0 mg, 0.169 mmol) in 4-methoxytoluene (1.0 mL). ^{1}H NMR (400 MHz, $CDCl_3$): δ 7.12 (d, $^3J_{HH}$ = 8.0 Hz, 2 H), 6.81 (d, $^3J_{HH}$ = 8.0 Hz, 2 H), 3.79 (s, 3 H), 2.25 (s, 2 H), 1.25 (s, 12 H). ^{11}B NMR (128 MHz, $CDCl_3$): δ 33.0. The ^{1}H and ^{11}B NMR data are similar to the reported data.[9]

2-(3-Chlorobenzyl)-4,4,5,5-tetramethyl-1,3,2-dioxaborolane.

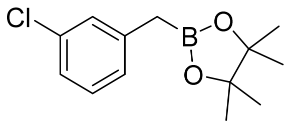

2-(3-Chlorobenzyl)-4,4,5,5-tetramethyl-1,3,2-dioxaborolane was prepared in 36% yield according to the general procedure using UiO-Co (1.0 mg, 0.2 mol % Co), B_2pin_2 (43.0 mg, 0.169 mmol) in 3-chlorotoluene (1.0 mL). ^{1}H NMR (400 MHz, $CDCl_3$): δ 7.18 (s, 1 H), 7.15 (t, $^3J_{HH}$ = 7.6 Hz, 1 H), 7.10 (d, $^3J_{HH}$ = 8.3 Hz, 1 H), 7.05 (d, $^3J_{HH}$ = 7.4 Hz, 1 H), 2.27 (s, 2 H), 1.23 (s, 12 H). ^{11}B NMR (128 MHz, $CDCl_3$): δ 32.7. The ^{1}H and ^{11}B NMR data are similar to the reported data.[9]

4,4,5,5-Tetramethyl-2-(1-phenylethyl)-1,3,2-dioxaborolane.

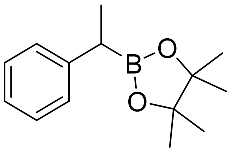

4,4,5,5-Tetramethyl-2-(1-phenylethyl)-1,3,2-dioxaborolane was prepared according to the general procedure using UiO-Co (1.0 mg, 0.2 mol % Co), B_2pin_2 (43.0 mg, 0.169 mmol) in ethylbenzene (2.0 mL). The crude product was a 78: 22 mixture of benzylic:aromatic boronate esters as detected by GC-MS. ^{1}H NMR (400 MHz, $CDCl_3$) of 4,4,5,5-Tetramethyl-2-(1-phenylethyl)-1,3,2-dioxaborolane: δ 7.26-7.20 (m, 4 H), 7.11 (m, 1 H), 2.42 (q, $^3J_{HH}$ = 7.5 Hz, 1 H), 1.30 (d, $^3J_{HH}$ = 7.5 Hz, 3 H), 1.19 (s, 6 H), 1.18 (s, 6 H). The ^{1}H NMR data is similar to the reported data.[11] Pure 1-phenylethanol was obtained in 70% yield after oxidation followed by silica gel column chromatography. ^{1}H NMR (400 MHz, $CDCl_3$) of 1-phenylethanol (CAS Number: 98-85-1): δ 7.3 – 7.21 (m, 5 H, C_6H_5), 4.75 (m, 1 H, C_6H_5CHMe), 1.40 (s, 3 H, (C_6H_5CHMe).

4,4,5,5-Tetramethyl-2-(2-phenylpropyl)-1,3,2-dioxaborolane.

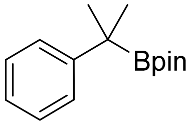

4,4,5,5-Tetramethyl-2-(2-phenylpropyl)-1,3,2-dioxaborolane was prepared according to the general procedure using UiO-Co (1.0 mg, 0.2 mol % Co), B_2pin_2 (43.0 mg, 0.169 mmol) in cumene (2.0 mL). The crude product was a 80: 20 mixture of benzylic:aromatic boronate esters as detected by GC-MS. ^{1}H NMR (400 MHz, $CDCl_3$) of 4,4,5,5-tetramethyl-2-(2-phenylpropyl)-1,3,2-dioxaborolane: δ 7.36-7.28 (m, 4 H), 7.17 (m, 1 H), 1.36 (s, 6 H), 1.22 (s, 12 H). The ^{1}H NMR data is similar to the reported data.[12] The crude product was directly oxidized and 2-phenyl-2-propanol obtained by silica gel column chromatography in 81% yield. ^{1}H NMR (400 MHz, $CDCl_3$) of 2-phenyl-2-propanol (CAS Number: 617-94-7): δ 7.51 (d, $^3J_{HH}$ = 8.0 Hz, 2 H), 7.37 (t, $^3J_{HH}$ = 8.0 Hz, 2 H), 7.27 (d, $^3J_{HH}$ = 8.0 Hz, 1 H), 1.60 (s, 6 H).

A typical procedure for catalytic benzylic C–H silylation followed by Tamao-Fleming oxidation to afford corresponding benzyl alcohol.

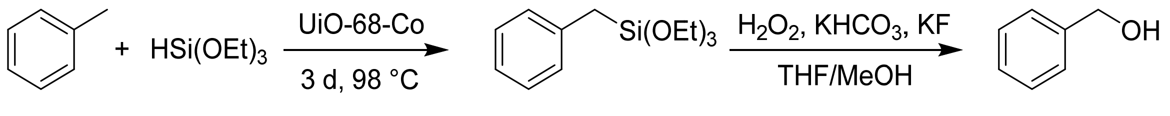

In a glovebox, UiO-CoCl (1.0 mg, 0.4 mol % Co) was charged into a small vial and 0.5 mL THF was added. Then, 15 μL $NaBEt_3H$ (1.0 M in THF) was added to the vial and the mixture was stirred slowly for 1 h in the glovebox. The solid was centrifuged out of suspension and washed with THF twice and then once with toluene. The solid suspended in 2 mL toluene was transferred to a Schlenk tube and $(EtO)_3SiH$ (32 μL, 0.17 mmol) was added to the mixture. The tube was heated under nitrogen at 98 °C for 3 d. The reaction mixture was cooled to room temperature and the solid was centrifuged out of suspension. The extract was concentrated *in vacuo* and then the residue was heated at 60 °C in vacuum for 3 h to give benzyltriethoxysilane as a colorless liquid in 89% yield (38 mg, 0.151 mmol). Attempts to isolate the silylated product by silica gel chromatograohy or preparative TLC were unsuccessful presumably due to the condensation reaction of the silylated product with silica. ^{1}H NMR (400 MHz, $CDCl_3$) of benzyltriethoxysilane: δ 7.27-7.19 (m, 4 H), 7.12 (t, $^3J_{HH}$ = 7.2 Hz, 1 H), 3.78 (q, $^3J_{HH}$ = 6.8 Hz, 6 H), 2.24 (s, 2 H), 1.19 (t, $^3J_{HH}$ = 6.8 Hz, 9 H). MS (CI^+) exact mass Calcd for $C_{13}H_{24}O_3Si$: m/z 128.0747 ($[(M + 2H)^{+2}]$), Found: 128.0734.

To the crude product mixture, $KHCO_3$ (102 mg, 1.0 mmol) and KF (59 mg, 1.0 mmol) in THF (1 mL) and MeOH (1 mL) was added H_2O_2 (30%, 0.3 mL) at 0 ºC. After being stirred for 24 h, the reaction was quenched by addition of saturated aqueous $Na_2S_2O_3$ (10 mL). The mixture was additionally stirred for 1 h and was extracted with EtOAc (3 × 6 mL). The extract was washed with aqueous NaCl (10 mL) and then dried over Na_2SO_4. After concentration, the residue was purified by column chromatography on silica gel with hexane–EtOAc as an eluent to give benzyl alcohol (15 mg, 0.138 mmol, 81% overall yield). ^{1}H NMR (400 MHz, $CDCl_3$) of benzyl alcohol (CAS Number: 100-51-6): δ 7.38-7.27 (m, 5 H, C_6H_5), 4.68 (s, 2 H, $PhCH_2Si$), 1.96 (br s, 1 H, OH).

4-Methylbenzyltriethoxysilane.

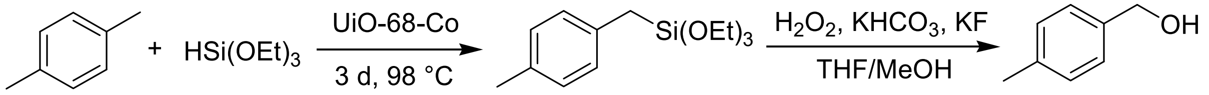

4-Methylbenzyltriethoxysilane was prepared according to the general procedure using UiO-Co (1.0 mg, 0.2 mol % Co), $HSi(OEt)_3$ (62 μL, 0.34 mmol) in *p*-xylene (2.0 mL). ^{1}H NMR (400 MHz, $CDCl_3$) of 4-methylbenzyltriethoxysilane: δ ^{1}H NMR (400 MHz, $CDCl_3$) of 4-methylbenzyltriethoxysilane: δ 7.18 (d, $^3J_{HH}$ = 8.4 Hz, 2 H, C_6H_4), 6.82 (d, $^3J_{HH}$ = 8.4 Hz, 2 H, C_6H_4), 3.81 (q, $^3J_{HH}$ = 6.8 Hz, 6 H), 2.31 (s, 3 H), 2.28 (s, 2 H), 1.21 (t, $^3J_{HH}$ = 6.8 Hz, 9 H). MS (ESI) exact mass Calcd for $C_{14}H_{24}O_3Si$: m/z 268.1495 ($[M^+]$), Found: 268.1476. 4-Methylbenzyltriethoxysilane was contaminated with impurities and could be purified by chromatography or distillation. The crude silane product was directly oxidized and pure 4-methylbenzyl alcohol was obtained as a white

solid in 70% yield after silica gel column chromatography. ^{1}H NMR (400 MHz, $CDCl_3$) of 4-methylbenzyl alcohol (CAS Number: 589-18-4): δ 7.27 (d, $^3J_{HH}$ = 8.0 Hz, 2 H, C_6H_4), 7.18 (d, $^3J_{HH}$ = 8.0 Hz, 2 H, C_6H_4), 4.66 (d, $^3J_{HH}$ = 6.0 Hz, 2 H, PhC*H*$_2$OH), 2.37 (s, 3 H, *Me*Ph), 1.66 (t, $^3J_{HH}$ = 6.0 Hz, 1 H, OH).

3,5-Dimethylbenzyl alcohol.

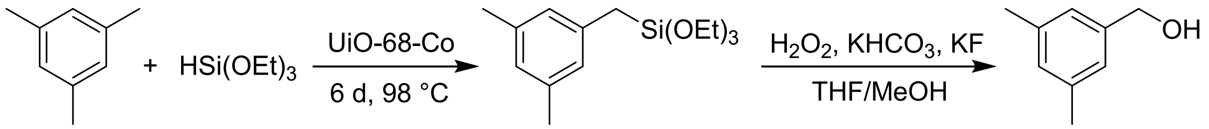

(3,5-Dimethylbenzyl)triethoxysilane was prepared according to the general procedure using UiO-Co (1.0 mg, 0.2 mol % Co), HSi(OEt)$_3$ (62 µL, 0.34 mmol) in mesitylene (2.0 mL). ^{1}H NMR (400 MHz, $CDCl_3$) of (3,5-dimethylbenzyl)triethoxysilane: δ 6.81 (s, 3 H), 3.86 (m, 6 H), 2.41 (s, 2 H), 2.29 (s, 6 H), 1.25 (m, 9 H). MS (CI$^+$) exact mass Calcd for $C_{15}H_{28}O_3Si$: m/z 142.0904 ([(M + 2H)$^{+2}$]), Found: 142.0904. (3,5-Dimethylbenzyl)triethoxysilane was contaminated with impurities and could be purified by chromatography or distillation. The crude silane product was directly oxidized and 3,5-dimethylbenzyl alcohol was obtained as a white solid in 80% yield after silica gel column chromatography. ^{1}H NMR (400 MHz, $CDCl_3$) of 3,5-dimethylbenzyl alcohol (CAS Number: 27129-87-9): δ 6.91 (s, 2 H, C_6H_3), 6.88 (s, 1 H, C_6H_3), 4.61 (d, $^3J_{HH}$ = 6.0 Hz, 2 H, PhC*H*$_2$OH), 2.35 (s, 6 H, *Me*Ph).

Triethoxy(1-phenylethyl)silane.

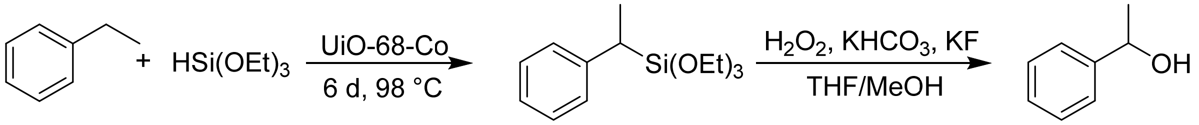

Triethoxy(1-phenylethyl)silane was prepared according to the general procedure using UiO-Co (1.0 mg, 0.2 mol % Co), HSi(OEt)$_3$ (62 µL, 0.34 mmol) in ethylbenzene (2.0 mL). ^{1}H NMR (400 MHz, $CDCl_3$) of triethoxy(1-phenylethyl)silane: δ ^{1}H NMR (400 MHz, $CDCl_3$) of triethoxy(1-phenylethyl)silane: δ 7.25-7.13 (m, 5 H), 3.76 (m, 6 H), 2.22 (m, 1 H), 1.34 (d, $^3J_{HH}$ = 7.2 Hz, 3 H), 1.20 (t, $^3J_{HH}$ = 6.8 Hz, 9 H). MS (CI$^+$) exact mass Calcd for $C_{14}H_{24}O_3Si$: m/z 268.1495 ([M$^+$]), Found: 268.1501. Triethoxy(1-phenylethyl)silane was contaminated with impurities and could be purified by chromatography or distillation. The crude product was directly oxidized and pure 1-phenylethanol was obtained as a white solid in 84% yield after oxidation followed by silica gel column chromatography. ^{1}H NMR (400 MHz, $CDCl_3$) of 1-phenylethanol (CAS Number: 98-85-1): δ 7.40-7.27 (m, 5 H, *Ph*CHMeOH), 4.91 (m, 1 H, PhC*H*MeOH), 1.89 (br s, 1 H, OH), 1.52 (d, $^3J_{HH}$ = 6.4 Hz, 3 H, PhCH*Me*OH).

4-Methoxybenzyltriethoxysilane.

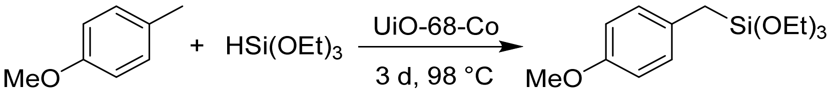

4-Methoxybenzyltriethoxysilane was prepared according to the typical procedure using UiO-Co (1.0 mg, 0.2 mol % Co), $HSi(OEt)_3$ (62 µL, 0.34 mmol) in 4-methylanisole (2.0 mL). ^1H NMR (400 MHz, $CDCl_3$) of 4-methoxybenzyltriethoxysilane: δ ^1H NMR (400 MHz, $CDCl_3$) of 4-methoxybenzyltriethoxysilane: δ 7.08 (d, $^3J_{HH}$ = 8.4 Hz, 2 H, C_6H_4), 6.80 (d, $^3J_{HH}$ = 8.4 Hz, 2 H, C_6H_4), 3.85 (m, 6 H, OCH_2CH_3), 2.28 (s, 2 H, CH_2Si), 1.22 (t, $^3J_{HH}$ = 6.8 Hz, 9 H, OCH_2CH_3). MS (ESI) exact mass Calcd for $C_{14}H_{24}O_4Si$: m/z 284.1444 ([M^+]), Found: 284.1452.

2-Phenyl-2-propanol.

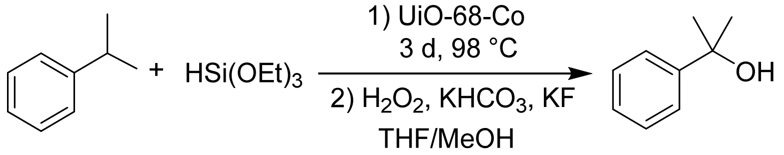

2-Phenyl-2-propanol was prepared according to the general procedure using UiO-Co (1.0 mg, 0.2 mol % Co), $HSi(OEt)_3$ (62 µL, 0.34 mmol) in cumene (2.0 mL). The product triethoxy(2-phenylpropan-2-yl)silane was contaminated with impurities and could be purified by chromatography or distillation. The crude silane product was directly oxidized and 2-phenyl-2-propanol was obtained in 61% yield after preparative TLC. ^1H NMR (400 MHz, $CDCl_3$) of 2-phenyl-2-propanol (CAS Number: 617-94-7): δ 7.45-7.26 (s, 5 H, C_6H_5), 1.55 (s, 6 H, $PhCMe_2OH$).

General procedure for UiO-Co catalyzed hydrogenation of olefins. In a nitrogen-filled glove box, UiO-CoCl (0.5 mg, 0.1 mol % Co) in 1.0 mL THF was charged into a glass vial. $NaBEt_3H$ (10 µL, 1.0 M in THF) was then added to the vial and the mixture was stirred for 1 h. The solid was then centrifuged, washed with THF twice, and transferred to a glass vial in 0.5 mL THF. The olefin substrate (0.34 mmol) was added to the vial. Then the vial was placed in a Parr reactor which was sealed under nitrogen atmosphere and charged with hydrogen to 40 bar. After stirring at room temperature for 12 h - 3 d, the pressure was released and the MOF catalyst was removed from the reaction mixture via centrifugation. Mesitylene (internal standard) was added to the organic extracts and the yield of the product was determined by integrations of the product and mesitylene peaks in the ^1H NMR spectra in $CDCl_3$.

A typical procedure for UiO-Co catalyzed hydrogenation of olefins.

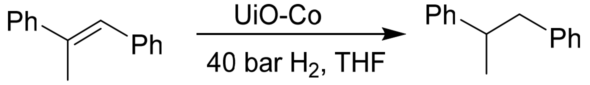

In a glovebox, UiO-CoCl in THF (0.5 mg, 0.1 mol% Co) was charged into a small vial and 0.5 mL THF was added. Then, 10 µL $NaBEt_3H$ (1.0 M in THF) was added to the vial and the mixture was stirred slowly for 1 h in the glovebox. The solid was centrifuged out of suspension and then washed with THF two times. Then, the black solid in 0.5 mL THF was transferred to a vial containing 0.5 mL THF solution of *trans*-α-methylstilbene (65.9 mg, 0.34 mmol). The vial was placed into a Parr pressure reactor in a nitrogen-filled glovebox. The reactor was then pressurized to 40 bar. After stirring at room temperature for 2 d, the solid was centrifuged out of suspension and extracted three

times with THF. The combined organic extracts were concentrated *in vacuo* to afford crude 1,2-diphenylpropane in quantitatively yield, which was sufficiently pure as shown in ^{1}H NMR spectrum (Supplementary Figure 39).

Reuse and recycle experiment procedure for UiO-Co-catalyzed hydrogenation of 1-octene.

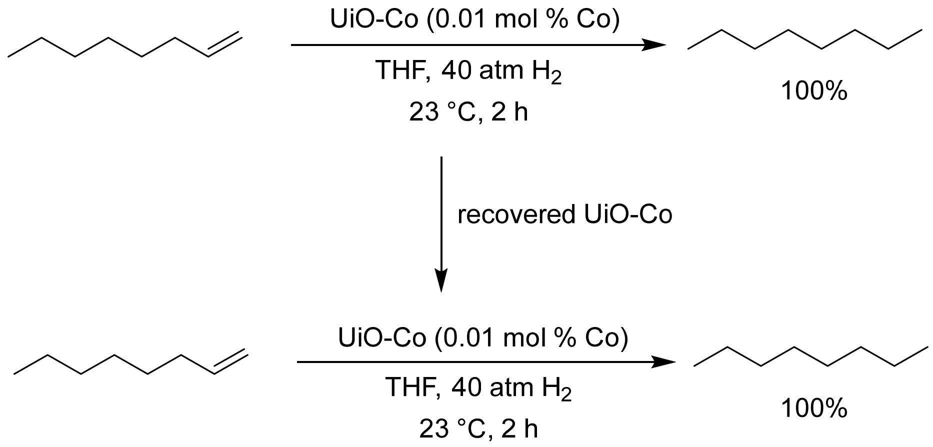

In a glovebox, a vial was charged with UiO-CoCl (2.0 mg, 0.01 mol % Co) in 1 mL THF. 20 μL $NaBEt_3H$ (1.0 M in THF) was added to the vial and the mixture was stirred slowly for 1 h in the glovebox. The solid was centrifuged out of suspension and washed with THF two times. Then, the solid in 1.0 mL THF was transferred to a vial and 1-octene (2 mL, 12.7 mmol) was added. The vial was placed into a Parr pressure reactor in a nitrogen-filled glovebox. The reactor was then pressurized to 40 bar. After 2 h, hydrogen was released and the solid was centrifuged out of suspension and extracted 2-3 times with THF in the glovebox. Quantitative yield of *n*-octane was obtained as determined by GC-MS and ^{1}H NMR with mesitylene as the internal standard.

The recovered solid catalyst was added to a vial containing 1-octene (2 mL, 12.7 mmol) in 1.0 mL THF. The vial was placed into a Parr pressure reactor in a nitrogen-filled glovebox. The reactor was then pressurized to 40 bar. After 16 h, the solid was centrifuged out of suspension and extracted 2-3 times with THF in the glovebox. *n*-Octane was obtained in quantitative yield as determined by GC-MS and ^{1}H NMR with mesitylene as the internal standard. UiO-Co was recovered and reused at least 16 times without loss of catalytic activity.

Procedures for UiO-Co-catalyzed hydroboration of carbonyl compounds.

In a glovebox, UiO-CoCl (1.0 mg, 0.01 mol % Co) was charged into a small vial and 0.5 mL THF was added. Then, 8 μL $NaBEt_3H$ (1.0 M in THF) was added to the vial and the mixture was stirred slowly for 1 h in the glovebox. The solid was centrifuged out of suspension and washed with THF two times. Then, THF was removed and the aldehyde or ketone (6.78 mmol) and pinacolborane (7.40 mmol) were added. The resultant mixture was transferred to a Schlenk tube and then heated at 60 °C outside of the glovebox and the progress of the reaction was monitored by GC. After complete conversion, the solid was centrifuged out of suspension and extracted with hexane 2-3 times. The combined organic extracts were concentrated *in vacuo* to yield the pure product.

A typical procedure for UiO-Co catalyzed hydroboration of ketones.

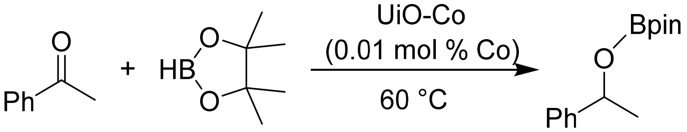

In a glovebox, UiO-CoCl (1.0 mg, 0.01 mol % Co) was charged into a small vial and 0.5 mL THF was added. Then, 8 µL $NaBEt_3H$ (1.0 M in THF) was added to the vial and the mixture was stirred slowly for 1 h in the glovebox. The solid was centrifuged out of suspension and washed with THF two times. Then, THF was removed and acetophenone (0.81 g, 6.78 mmol) and pinacolborane (0.95 g, 7.40 mmol) was added. The resultant mixture was transferred to a Schlenk tube and then heated at 60 °C outside of the glovebox for 2 d. Then, the solid was centrifuged out of suspension and extracted with hexane 2-3 times. The combined organic extracts were concentrated *in vacuo* to yield 4,4,5,5-Tetramethyl-2-(1-phenylethoxy)-1,3,2-dioxaborolane as a colorless oil (1.63 g, 6.57 mmol, 96.9%). The crude borate ester was sufficiently pure for further uses as shown by ^{1}H NMR spectrum (Supplementary Figure 41). ^{1}H NMR ($CDCl_3$, 400 MHz): δ 7.40-7.25 (m, 5 H, C_6H_5), 5.27 (q, $^3J_{HH}$ = 6.4 Hz, 1 H, OC*H*Me) 1.52 (d, $^3J_{HH}$ = 8.0 Hz, 3 H, OCH*Me*), 1.27 (s, 6 H, $BO_2C_2Me_4$), 1.24 (s, 6 H, $BO_2C_2Me_4$). ^{11}B NMR ($CDCl_3$, 128 MHz): δ 22.5. The ^{1}H and ^{11}B NMR data are similar to the reported data.[13]

4,4,5,5-Tetramethyl-2-((4-methylbenzyl)oxy)-1,3,2-dioxaborolane.[14]

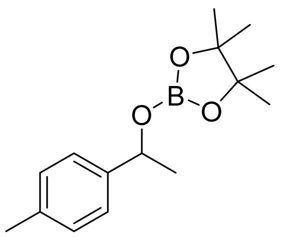

4,4,5,5-Tetramethyl-2-((4-methylbenzyl)oxy)-1,3,2-dioxaborolane was prepared in 96% yield according to the general procedure using UiO-Co (1.0 mg, 0.01 mol % Co), 4-methyl-acetophenone (0.91 g, 6.78 mmol) and pinacolborane (0.95 g, 7.40 mmol). ^{1}H NMR (400 MHz, C_6D_6): δ 7.11-6.92 (m, 4 H, C_6H_4), 5.29 (m, H, OC*H*Me), 2.07 (s, 3 H, ArCH_3) 1.47 (d, 3 H, OCH*Me*), 1.02 (s, 12 H, $BO_2C_2Me_4$). ^{11}B NMR ($CDCl_3$, 128 MHz): δ 22.4.

4,4,5,5-Tetramethyl-2-((4-methoxybenzyl)oxy)-1,3,2-dioxaborolane.[15]

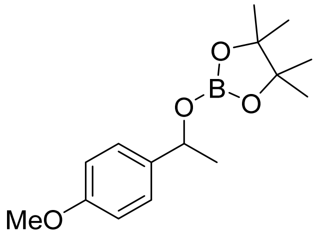

4,4,5,5-Tetramethyl-2-((4-methoxybenzyl)oxy)-1,3,2-dioxaborolane was prepared in quantitative yield according to the general procedure using UiO-Co (1.0 mg, 0.01 mol % Co), 4-methoxy-acetophenone (1.02 g, 6.78 mmol) and

pinacolborane (0.95 g, 7.40 mmol). ^{1}H NMR ($CDCl_3$, 400 MHz): δ 7.30 (d, $^3J_{HH}$ = 8.0 Hz, 2 H, C_6H_4), 6.87 (d, $^3J_{HH}$ = 8.0 Hz, 2 H, C_6H_4), 5.21 (q, $^3J_{HH}$ = 6.4 Hz, 1 H, OC*H*Me), 3.80 (s, OCH_3), 1.48 (d, $^3J_{HH}$ = 6.4 Hz, 3 H, OCH*Me*), 1.25 (s, 6 H, $BO_2C_2Me_4$), 1.22 (s, 6 H, $BO_2C_2Me_4$). ^{11}B NMR ($CDCl_3$, 128 MHz): δ 22.2.

2-(*sec*-Butoxy)-4,4,5,5-tetramethyl-1,3,2-dioxaborolane.[16]

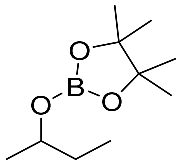

2-(*sec*-Butoxy)-4,4,5,5-tetramethyl-1,3,2-dioxaborolane was prepared in 86% yield according to the general procedure using UiO-Co (1.0 mg, 0.01 mol % Co), butan-2-one (0.489 g, 6.78 mmol) and pinacolborane (0.95 g, 7.40 mmol). ^{1}H NMR ($CDCl_3$, 400 MHz): δ 4.08 (m, 1 H, OC*H*), 1.49 (m, 2 H, OCHCH_2Me), 1.17 (d, $^3J_{HH}$ = 6.0 Hz, 3 H, OCH*Me*), 1.07 (s, 12 H, $BO_2C_2Me_4$), 0.88 (t, $^3J_{HH}$ = 7.4 Hz, 3 H, CH_2Me). ^{11}B NMR ($CDCl_3$, 128 MHz): δ 22.9.

2-(Benzyloxy)-4,4,5,5-tetramethyl-1,3,2-dioxaborolane.[13]

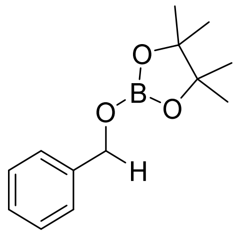

2-(Benzyloxy)-4,4,5,5-tetramethyl-1,3,2-dioxaborolane was prepared in 98% yield according to the general procedure using UiO-Co (1.0 mg, 0.01 mol % Co), benzaldehyde (0.719 g, 6.78 mmol) and pinacolborane (0.95 g, 7.40 mmol). ^{1}H NMR (C_6D_6, 600 MHz): δ 7.31 (d, $^3J_{HH}$ = 7.4 Hz, 2 H, *ortho*-C_6H_5), 7.14 (t, $^3J_{HH}$ = 7.3 Hz, 2 H, *meta*-C_6H_5), 7.04 (t, $^3J_{HH}$ = 7.1 Hz, 1 H, *para*-C_6H_5), 4.96 (s, 2 H, OCH_2), 1.05 (s, 12 H, $BO_2C_2Me_4$). ^{11}B NMR ($CDCl_3$, 128 MHz): δ 22.8.

GC analysis. The conversions of reactions and yields of the products were determined by gas chromatography (GC) using a Shimadzu GC-2010 gas chromatograph equipped with a flame ionization detector (FID) and Supelco β-dex 120 column.

GC conditions: Inj: 220 °C; Det: 250 °C; Column temp: 80 °C followed by a ramp of 2 °C/min to 200 °C and held for 10 minutes; Column flow: 1.11 mL/min.

General Procedures for UiO-Co-catalyzed alkene hydroboration

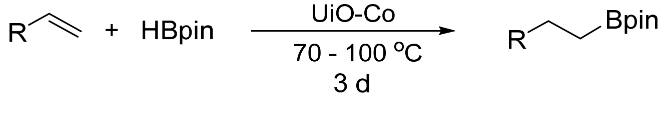

In a glovebox, UiO-CoCl (10 mg, 0.2 mol% Co) was charged into a small vial and 0.5 mL THF was added. Then, 8 μL $NaBEt_3H$ (1.0 M in THF) was added to the vial and the mixture was stirred slowly for 1 h in the glovebox. The solid was centrifuged out of suspension and washed with THF two times. Then, THF was removed and alkene (3.39 mmol) and pinacolborane (5.08 mmol) were added. The resultant mixture was transferred to a Schlenk tube and then heated at 100 °C outside of the glovebox and the progress of the reaction was monitored by GC. After complete conversion, the solid was centrifuged out of suspension and extracted with hexane 2-3 times. The combined organic extracts were concentrated *in vacuo* to yield the pure product.

A typical procedure of catalytic alkene hydroboration

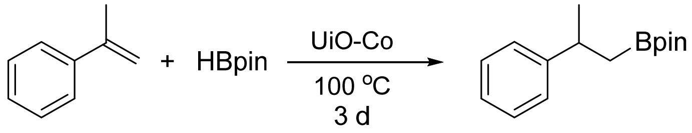

In a glovebox, UiO-CoCl (8 μmol Co) was charged into a small vial and 0.5 mL THF was added. Then, 80 μL $NaBEt_3H$ (1.0 M in THF) was added to the vial and the mixture was stirred slowly for 1 h in the glovebox. The solid was centrifuged out of suspension and washed with THF two times. Then, THF was removed and α-methylstyrene (0.26 mL, 2.0 mmol) and pinacolborane (0.35 mL, 3.0 mmol) were added. The resultant mixture was transferred to a Schlenk tube and then heated at 100 °C outside of the glovebox for 3 days. After complete conversion, the reaction mixture was filtered through Celite and washed with dichloromethane. The filtrate was concentrated *in vacuo* to yield the pure product (456 mg, 1.85 mmol, 93%). ^{1}H NMR of the obtained crude product was sufficiently pure 4,4,5,5-tetramethyl-2-(2-phenylpropyl)-1,3,2-dioxaborolane as shown in Supplementary Figure 42. ^{1}H NMR (500 MHz, Chloroform-*d*) δ 7.28 – 7.22 (m, 4 H), 7.18 – 7.11 (m, 1 H), 3.03 (h, $^3J_{HH}$ = 7.2 Hz, 1 H), 1.30 – 1.25 (m, 5 H), 1.16 (s, 12 H). ^{11}B NMR ($CDCl_3$, 128 MHz): δ 33.5. The ^{1}H and ^{11}B NMR data are similar to the reported data.[17]

4,4,5,5-tetramethyl-2-phenethyl-1,3,2-dioxaborolane

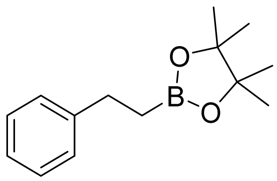

4,4,5,5-tetramethyl-2-phenethyl-1,3,2-dioxaborolane was prepared in 93% yield according to the general procedure using UiO-Co (2.0 μmol Co), styrene (0.23 mL, 2.0 mmol) and pinacolborane (0.35 mL, 3.0 mmol). ^{1}H NMR (500 MHz, Chloroform-*d*) δ 7.28 – 7.20 (m, 4 H), 7.15 (t, $^3J_{HH}$ = 7.1 Hz, 1 H), 2.77 – 2.73 (m, 2 H), 1.22 (s, 12 H), 1.17 – 1.13 (m, 2 H). ^{11}B NMR ($CDCl_3$, 128 MHz): δ 33.7. The ^{1}H and ^{11}B NMR data are similar to the reported data.[17]

4,4,5,5-tetramethyl-2-octyl-1,3,2-dioxaborolane

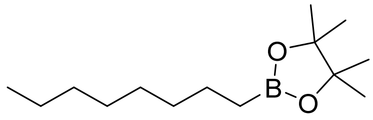

4,4,5,5-tetramethyl-2-octyl-1,3,2-dioxaborolane was prepared in 61% yield according to the general procedure using UiO-Co (8 μmol Co), 1-octene (0.31 mL, 2.0 mmol) and pinacolborane (0.35 mL, 3.0 mmol). ^{1}H NMR (500 MHz, Chloroform-*d*) δ 1.39 (p, $^3J_{HH}$ = 7.0 Hz, 2 H), 1.31 – 1.25 (m, 10 H), 1.24 (s, 12 H), 0.87 (t, $^3J_{HH}$ = 6.9 Hz, 3 H), 0.76 (t, $^3J_{HH}$ = 7.8 Hz, 2 H). ^{11}B NMR ($CDCl_3$, 128 MHz): δ 33.9. The ^{1}H and ^{11}B NMR data are similar to the reported data.[17]

General procedures for UiO-Fe catalyzed C–H amination

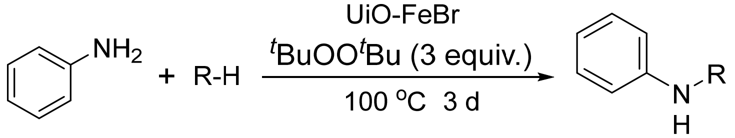

In a glovebox, UiO-FeBr (10 μmol Fe) was centrifuged off THF, washed with heptane twice and transferred into a Schlenk tube with C–H substrate (3 mmol), then aniline (0.1 mmol) and ditertbutylperoxide (0.4 mmol) were directly added to the Schlenk tube. The Schlenk tube was stirred at 100 °C until no aniline was observed by GC-MS analysis. Then, the solid was centrifuged out of suspension and washed with THF two times, and the extract concentrated under rotavap. Nitromethane (internal standard) was added to the reaction mixture, and the yield of the product was determined by integrations of the product and nitromethane peaks in the ^{1}H NMR spectra in $CDCl_3$.

N-phenyl-2,3-dihydro-1H-inden-1-amine

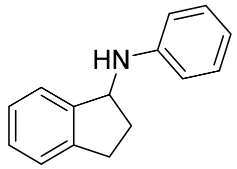

In a glovebox, UiO-FeBr (16 μmol Fe) was centrifuged off THF, washed with heptane twice and transferred into a Schlenk tube with indane (0.587 mL, 4.8 mmol). Aniline (14.6 μL, 0.16 mmol) and ditertbutylperoxide (88.2μL, 0.48 mmol) were directly added to the Schlenk tube before it was stirred at 100 °C for 5 days. Then, the solid was centrifuged out of suspension and washed with THF two times, and the extract concentrated under rotavap. The residue was purified by column chromatography on silica gel with 1% Et_3N and 5% EtOAc in hexane to afford *N*-phenyl-2,3-dihydro-1H-inden-1-amine (16 mg, 76 μmol, 53%). ^{1}H NMR (500 MHz, Chloroform-*d*) δ 7.41 (d, $^3J_{HH}$ = 7.4 Hz, 1 H), 7.32 – 7.27 (m, 3 H), 7.27 – 7.22 (m, 2 H), 6.77 (t, $^3J_{HH}$ = 8.2 Hz, 3H), 5.05 (t, $^3J_{HH}$ = 6.7 Hz, 1 H), 3.94 (s, 1 H), 3.06 (ddd, *J* = 15.8, 8.6, 4.3 Hz, 1 H), 2.93 (dt, *J* = 15.9, 7.9 Hz, 1 H), 2.69 – 2.54 (m, 1 H), 1.95 (dt, *J* = 14.9, 7.7 Hz, 1 H). The ^{1}H data is similar to the reported data.[18]

N-Cyclohexyl-1,2,3,4-tetrahydronaphthalen-1-amine

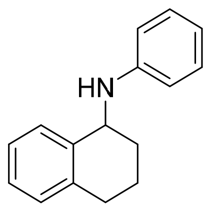

N-Cyclohexyl-1,2,3,4-tetrahydronaphthalen-1-amine was prepared in 49% yield according to the general procedure using UiO-FeBr (16 μmol Fe), tetraline (0.436 mL, 3.2 mmol), $(^tBuO)_2$ (88.2μL, 0.48 mmol) and aniline (14.6 μL, 0.16 mmol). ^{1}H NMR (500 MHz, Chloroform-*d*) δ 7.41 (dd, *J* = 7.3, 1.8 Hz, 1 H), 7.23 – 7.15 (m, 4 H), 7.13 (dd, *J* = 7.4, 1.8 Hz, 1 H), 6.71 (t, *J* = 7.4 Hz, 1 H), 6.68 (d, *J* = 7.6 Hz, 2 H), 4.64 (s, 1 H), 3.88 (s, 1 H), 2.89 – 2.72 (m, 2 H), 2.02 – 1.94 (m, 2 H), 1.93 – 1.74 (m, 2 H). The ^{1}H data is similar to the reported data.[18]

N-(Cyclohex-2-en-1-yl)aniline

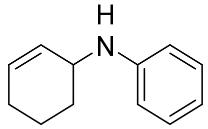

N-cyclohexyl-1,2,3,4-tetrahydronaphthalen-1-amine was prepared in 41% yield according to the general procedure using UiO-FeBr (4 μmol Fe), cyclohexene (0.500 mL, 5 mmol), $(^tBuO)_2$ (73.5 μL, 0.40 mmol) and aniline (18.3 μL, 0.20 mmol). ^{1}H NMR (500 MHz, Chloroform-*d*) δ 7.21 – 7.14 (m, 2 H), 6.82 – 6.65 (m, 3 H), 5.93 – 5.83 (m, 1 H), 5.79 – 5.72 (m, 1 H), 3.99 (br, 1 H), 3.62 (br, 1 H), 2.06 – 1.99 (m, 2 H), 1.95 – 1.87 (m, 1 H), 1.67 – 1.62 (m, 3 H). The ^{1}H data is similar to the reported data.[18]

Supplementary References

1. Campbell, T.W. Dicarboxylation of terphenyl. *Journal of the American Chemical Society* **82**, 3126-3128 (1960).
2. Cavka, J.H.*, et al.* A new zirconium inorganic building brick forming metal organic frameworks with exceptional stability. *Journal of the American Chemical Society* **130**, 13850-13851 (2008).
3. Kickelbick, G., Wiede, P. & Schubert, U. Variations in capping the Zr6O4(OH)4 cluster core: X-ray structure analyses of [Zr6(OH)4O4(OOC–CH=CH2)10]2(μ-OOC–CH=CH2)4 and Zr6(OH)4O4(OOCR)12(PrOH) (R = Ph, CMe = CH2). *Inorg Chim Acta* **284**, 1-7 (1999).
4. Sheldrick, G. Crystal structure refinement with SHELXL. *Acta Crystallographica Section C* **71**, 3-8 (2015).
5. Dolomanov, O.V., Bourhis, L.J., Gildea, R.J., Howard, J.A.K. & Puschmann, H. OLEX2: a complete structure solution, refinement and analysis program. *Journal of Applied Crystallography* **42**, 339-341 (2009).
6. Sheldrick, G. A short history of SHELX. *Acta Crystallographica Section A* **64**, 112-122 (2008).
7. Rehr, J.J. & Albers, R.C. Theoretical approaches to x-ray absorption fine structure. *Reviews of Modern Physics* **72**, 621-654 (2000).
8. Ravel, B. & Newville, M. ATHENA, ARTEMIS, HEPHAESTUS: data analysis for X-ray absorption spectroscopy using IFEFFIT. *Journal of Synchrotron Radiation* **12**, 537-541 (2005).

9. Larsen, M.A., Wilson, C.V. & Hartwig, J.F. Iridium-Catalyzed Borylation of Primary Benzylic C–H Bonds without a Directing Group: Scope, Mechanism, and Origins of Selectivity. *Journal of the American Chemical Society* **137**, 8633-8643 (2015).
10. Boebel, T.A. & Hartwig, J.F. Iridium-Catalyzed Preparation of Silylboranes by Silane Borylation and Their Use in the Catalytic Borylation of Arenes. *Organometallics* **27**, 6013-6019 (2008).
11. Dudnik, A.S. & Fu, G.C. Nickel-Catalyzed Coupling Reactions of Alkyl Electrophiles, Including Unactivated Tertiary Halides, To Generate Carbon–Boron Bonds. *Journal of the American Chemical Society* **134**, 10693-10697 (2012).
12. Glasspoole, B.W., Ghozati, K., Moir, J.W. & Crudden, C.M. Suzuki-Miyaura cross-couplings of secondary allylic boronic esters. *Chemical Communications* **48**, 1230-1232 (2012).
13. Arrowsmith, M., Hadlington, T.J., Hill, M.S. & Kociok-Kohn, G. Magnesium-catalysed hydroboration of aldehydes and ketones. *Chemical Communications* **48**, 4567-4569 (2012).
14. Yang, Z.*, et al.* An Aluminum Hydride That Functions like a Transition-Metal Catalyst. *Angewandte Chemie International Edition* **54**, 10225-10229 (2015).
15. Hadlington, T.J., Hermann, M., Frenking, G. & Jones, C. Low Coordinate Germanium(II) and Tin(II) Hydride Complexes: Efficient Catalysts for the Hydroboration of Carbonyl Compounds. *Journal of the American Chemical Society* **136**, 3028-3031 (2014).
16. Eedugurala, N.*, et al.* Mesoporous Silica-Supported Amidozirconium-Catalyzed Carbonyl Hydroboration. *ACS Catalysis* **5**, 7399-7414 (2015).
17. Zhang, L., Peng, D., Leng, X. & Huang, Z. Iron-Catalyzed, Atom-Economical, Chemo-and Regioselective Alkene Hydroboration with Pinacolborane. *Angewandte Chemie International Edition* **52**, 3676-3680 (2013).
18. Gephart, R.T.*, et al.* Catalytic C-H Amination with Aromatic Amines. *Angewandte Chemie* **124**, 6594-6598 (2012).
